# Tunable High‐Performance Photo‐Assisted Li–O_2_ Batteries by the Construction of Ferroelectric Photocathode

**DOI:** 10.1002/advs.202414616

**Published:** 2025-05-05

**Authors:** Huan‐Feng Wang, Yu‐Fei Wang, De‐Hui Guan, Xiao‐Xue Wang, Xin‐Yue Ma, Xin‐Yuan Yuan, Ji‐Jing Xu

**Affiliations:** ^1^ College of Food and Chemical Engineering Zhengzhou Key Laboratory of Functional Electrocatalysis and Chemical Energy Storage Zhengzhou University of Technology Zhengzhou 450044 P. R. China; ^2^ State Key Laboratory of Inorganic Synthesis and Preparative Chemistry College of Chemistry Jilin University Changchun 130012 P. R. China; ^3^ International Center of Future Science Jilin University Changchun 130012 P. R. China

**Keywords:** charge separation, ferroelectric photocatalyst, internal electric fields, Li–O_2_ batteries, photo‐assisted

## Abstract

Photo‐assistance is considered to be an effective approach to reducing the overpotential for lithium‐oxygen (Li–O_2_) batteries. However, the advancement is greatly hindered by the rapidly recombined photoexcited electrons and holes upon the discharging and charging processes. Herein, a breakthrough in overcoming these challenges is achieved by developing an efficient ferroelectric photocatalyst with spontaneous polarization‐induced internal electric fields. Tungsten (W) doped Bi_3_TiNbO_9_ (Bi_3_TiNbO_9_‐W) as a photocatalyst exhibits enhanced anisotropic migration of photogenerated electrons and holes, which play a key role in reducing the overpotential in the discharge and charge processes, enabling the desirable spatial separation of carriers. Benefiting from the driving force for charge separation, the photocatalytic oxygen reduction and evolution reaction activity is largely improved. As a result, the Bi_3_TiNbO_9_‐W‐based Li–O_2_ batteries have shown incremental round‐trip efficiencies of 95.9% based on the ultra‐high discharge voltage (3.25 V) and ultra‐low charge voltage (3.39 V). Besides, the constructed photo‐assisted Li–O_2_ batteries deliver a high rate capability and ultralong durability within 960 h. These findings demonstrate the crucial role of ferroelectric polarization in the improved photocatalytic reaction process, providing significant insight into addressing the overpotential bottleneck in Li–O_2_ batteries.

## Graphical abstract

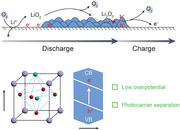

## 1 Introduction

**Figure 1:**
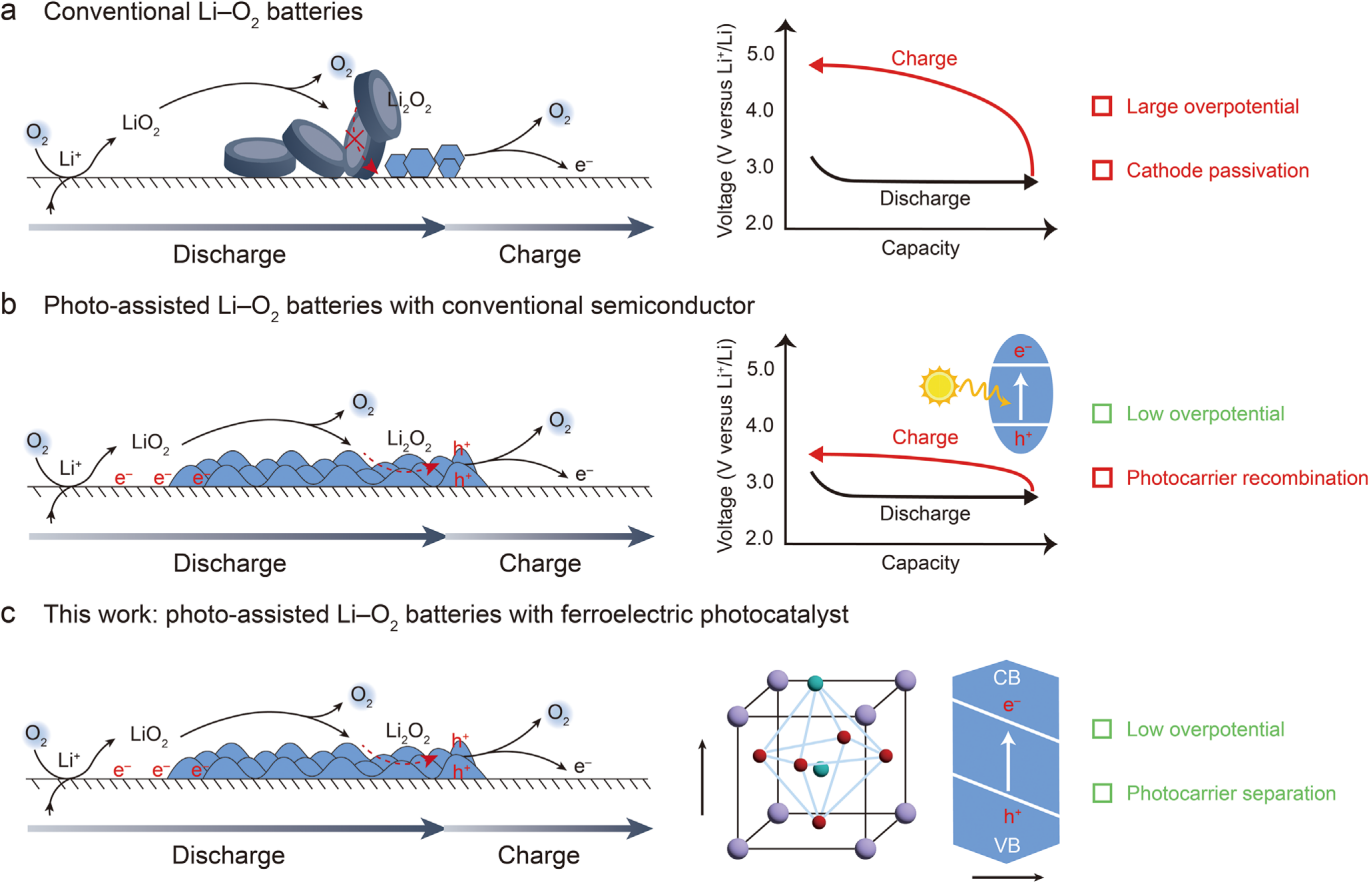
Illustration on the reaction process and corresponding challenges of a) conventional Li–O_2_ batteries and b) photo‐assisted Li–O_2_ batteries. c) Illustration on the photo‐assisted Li–O_2_ batteries developed with a ferroelectric photocatalyst in this work.

Lithium‐oxygen (Li–O_2_) batteries are regarded as the ultimate energy storage technology owing to their exceptionally theoretical gravimetric energy density of 3500 Wh kg^−1^.^[^ ^1^, ^2^, ^3^ ^]^ The performance of Li–O_2_ batteries is primarily influenced by the formation and decomposition of discharge products during the oxygen reduction reaction (ORR) and oxygen evolution reaction (OER) processes, respectively.^[^ ^4^, ^5^, ^6^ ^]^ However, the sluggish ORR and OER reactions kinetics caused by the insulated discharge products always lead to high overpotential during the discharge and charge process and poor cycle life (**Figure** **1**a), hindering the practical application of Li–O_2_ batteries.^[^ ^7^, ^8^ ^]^ Despite numerous cathode catalysts such as noble metals, transitional metal oxides, single‐atom catalysts, etc., having been dedicated to improving the ORR and OER kinetics, large discharge/charge polarization (≈1.0 V) remains.^[^ ^9^, ^10^, ^11^ ^]^

Recently, optical fields have been introduced to address the challenge of high overpotential in Li–O_2_ battery systems and other energy conversion/storage devices.^[^ ^12^, ^13^, ^14^ ^]^ A key factor of this technology is the selection of a proper semiconductor capable of separating electrons and holes upon illumination, with suitable energy levels for the ORR and OER processes.^[^ ^15^, ^16^, ^17^ ^]^ The lifetime of photoelectrons and holes located in the conduction and valence bands (CB and VB) of semiconductor photocatalysts has to be aligned with the reaction kinetics of ORR or OER, accelerating the cathode reactions in Li–O_2_ battery systems.^[^ ^18^ ^]^ Semiconductor photocatalysts such as C_3_N_4_, TiO_2_, and Fe_2_O_3_ have displayed significant progress in utilizing light to promote the ORR/OER processes.^[^ ^19^ ^]^ However, many commonly used semiconductor materials primarily absorb the ultraviolet (UV) light, limiting the utilization of solar energy (Figure 1b). Additionally, they suffer from the severe recombination of photoelectron‐hole pairs and the induced mismatch between carrier lifetime and O_2_ redox kinetics at cathodes.^[^ ^20^ ^]^ Rationally designing semiconductor cathode photocatalysts with enhanced redox capabilities of carriers, effective charge separation, and broad light absorption is crucial to improving the performance of photo‐assisted Li–O_2_ batteries.

Constructing heterojunction systems, such as type‐II heterojunctions, p‐n junctions, and Schottky junctions, has been employed to suppress the rapid electron–hole recombination and extend light absorption by aligning electronic bands.^[^ ^21^, ^22^, ^23^ ^]^ Yet, the synthetic methods for heterostructures are complicated and rigorous.^[^ ^24^ ^]^ Recently, ferroelectric materials have attracted wide attention in the field of photocatalysis.^[^ ^25^, ^26^ ^]^ In ferroelectric semiconductors, a built‐in electric field can be realized in single‐crystal semiconductors utilizing atomic‐level spontaneous symmetry breaking, heteroatom‐induced lattice distorting, or external stress‐caused strain gradient.^[^ ^27^, ^28^ ^]^ The built‐in electric field of semiconductors usually induces the formation of different charged polar surfaces (Figure 1c). Driven by the built‐in electric field, the photocarriers can be spatially separated from the bulk to different surfaces over these materials during the solar energy conversion.^[^ ^29^, ^30^ ^]^ Hence, it is of great significance to design highly efficient photocatalysts for photo‐assisted Li–O_2_ batteries using ferroelectric materials.

Bi_3_TiNbO_9_ is an Aurivillius‐type layered ferroelectric photocatalyst with a structural distortion‐induced depolarization field, which has also been demonstrated to display favorable potential in photocatalytic reactions.^[^ ^31^, ^32^ ^]^ Nevertheless, the nature of poor interlayer charge transport has greatly hindered the photoelectric performance of Bi_3_TiNbO_9_.^[^ ^33^ ^]^ Heteroatom‐induced lattice distorting is considered to be an effective solution to overcome the abovementioned limitations.^[^ ^34^ ^]^ In this work, tungsten (W) element doping Bi_3_TiNbO_9_ (Bi_3_TiNbO_9_‐W) was employed as functional cathode in a photo‐assisted Li–O_2_ battery system, taking advantage of its superior light harvesting capability and electron–hole separation rate. Spectroscopy technologies and density functional theory calculations reveal that the built‐in electric field and W doping induce the charge transfer. This spatially separated photoelectrons and holes exhibits strong redox capabilities, remarkably promoting the ORR and OER kinetics. As a result, the photo‐assisted Li–O_2_ battery with Bi_3_TiNbO_9_‐W cathode displayed an ultralow overpotential of 0.14 V at a current density of 0.04 mA cm^−2^ and maintained high cyclic stability within 960 h. The functional‐oriented design in this work demonstrates to be an effective utilization of solar energy to improve the round‐trip efficiency of Li–O_2_ batteries by regulating the crystal structure and electronic structure arising from element doping in ferroelectric materials.

## 2 Results and Discussion

### 2.1 Design and Characterization of the Ferroelectric Photocatalyst

**Figure 2:**
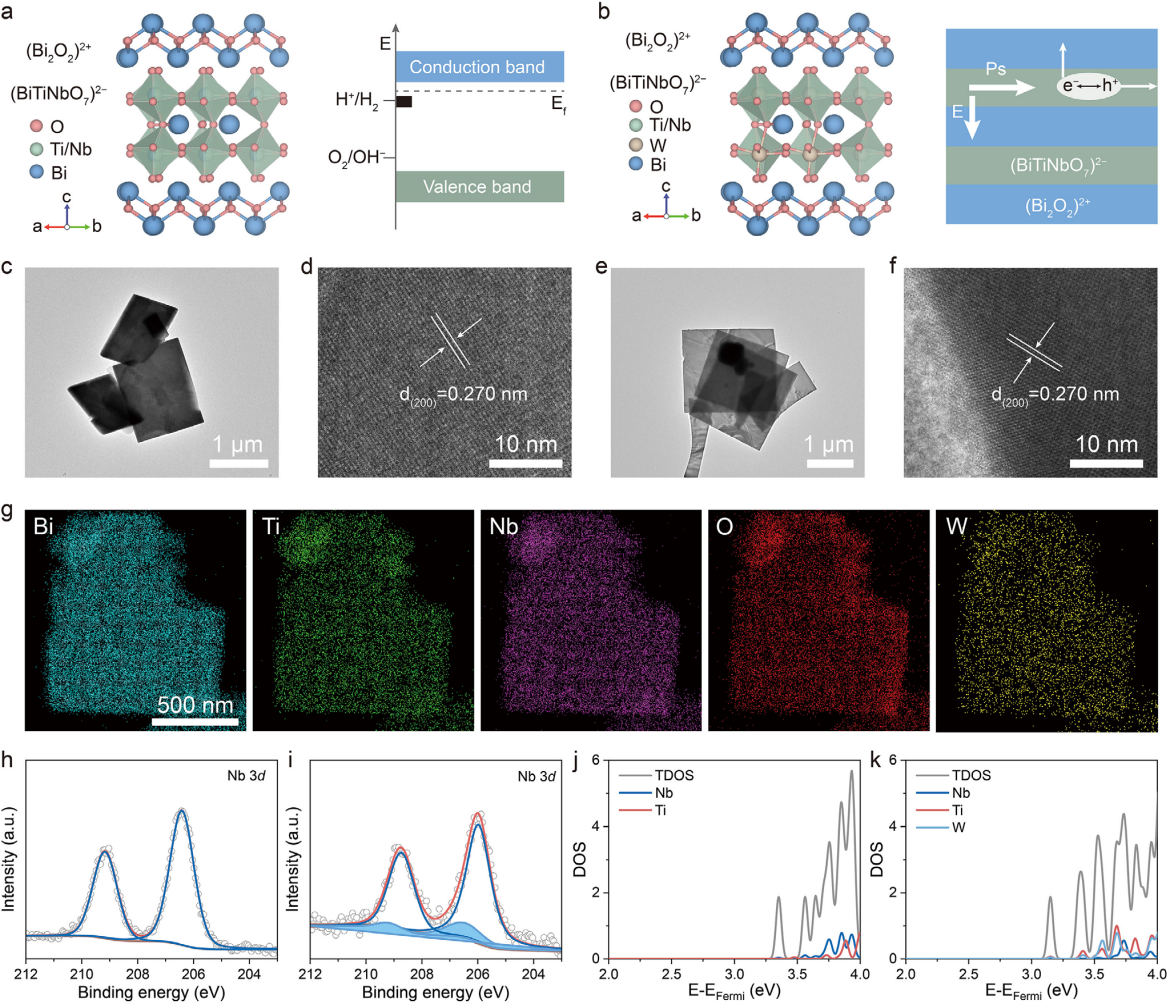
a) Crystal structure of Bi_3_TiNbO_9_ and its band structure referring to the potentials for water splitting and Bi^3+^ reduction. b) Crystal structure of Bi_3_TiNbO_9_‐W and its band structure referring to the potentials for water splitting and Bi^3+^ reduction. c,d) TEM and HRTEM images of Bi_3_TiNbO_9_. e,f) TEM and HRTEM images of Bi_3_TiNbO_9_‐W. g) EDS elemental mapping images of Bi_3_TiNbO_9_‐W. Deconvolved XPS spectra of Nb 3*d* of h) Bi_3_TiNbO_9_ and i) Bi_3_TiNbO_9_‐W. The calculated total density of states (TDOS) and projected density of states (PDOS) of j) Bi_3_TiNbO_9_ and (k) Bi_3_TiNbO_9_‐W.

Bi_3_TiNbO_9_ is a layered compound composed of alternating (Bi_2_O_2_)^2+^ layer and (BiTiNbO_7_)^2−^ layers along the c axis,^[^ ^35^ ^]^ with its conduction band minimum (CBM) and valence band maximum (VBM) spanning the hydrogen and oxygen evolution potentials, making it a promising candidate for photocatalytic overall water splitting (**Figure** **2**a). However, the slow migration of photogenerated electrons along the c‐axis increases the recombination probability of the photogenerated carriers due to a significant interlayer barrier. By doping with W elements, an additional built‐in electric field was introduced, perpendicularly oriented to the depolarization field in Bi_3_TiNbO_9_ nanosheets (Bi_3_TiNbO_9_‐W). And this modification is expected to overcome the potential barrier between the (Bi_2_O_2_)^2+^ and (BiTiNbO_7_)^2−^ layers (Figure 2b). Furthermore, the W dopant could strengthen the structural distortion, thereby facilitating the anisotropic flow of the photogenerated carriers. Benefiting from the unique advantages of Bi_3_TiNbO_9_‐W, excellent battery performance is anticipated to be achieved by employing it as the cathode catalyst for photo‐assisted Li–O_2_ batteries.

Bi_3_TiNbO_9_ and Bi_3_TiNbO_9_‐W were prepared via a conventional solid‐state flux method, respectively. Scanning electron microscopy (SEM) and transmission electron microscopy (TEM) were used to characterize the structure and morphology of the samples. According to the SEM and TEM results, Bi_3_TiNbO_9_ and Bi_3_TiNbO_9_‐W nanosheets with an average lateral width of ≈1 µm (Figure 2c,e; Figure S1, Supporting Information) can be observed, indicating a negligible effect of W doping on the morphology of the target product. The X‐ray diffraction (XRD) characterization patterns of both Bi_3_TiNbO_9_ and Bi_3_TiNbO_9_‐W closely match the standard card (PDF#39‐0233, space group: A21am) for Bi_3_TiN_b_O_9_ (Figure S2a, Supporting Information). The relative intensity variations in the diffraction peaks at 14 and 24°, corresponding to the (001) and (110) planes of Bi_3_TiNbO_9_, are observed. Raman spectra, with an excitation wavelength of 532 nm, were carried out to examine the bonding environment and structure of the Bi_3_TiNbO_9_‐W nanosheet (Figure S2b, Supporting Information). The *E_g_* modes, associated with the stretching of diagonal oxygen ions on the ab plane, split into *B_2g_* at 533 cm^−1^ and *B_3g_* at 573 cm^−1^, which can be attributed to the orthogonal distortion of Bi_3_TiNbO_9_. Further microstructural identification was studied by high‐resolution TEM (HRTEM). As shown in Figure 2d,f, the lattice fringes of d = 0.270 nm can be indexed as the (200) crystal plane of Bi_3_TiNbO_9_ and Bi_3_TiNbO_9_‐W, consistent with the XRD results. The distribution of W elements in Bi_3_TiNbO_9_‐W nanosheets was confirmed by energy‐dispersive X‐ray spectroscopy (EDS). The elements Bi, Ti, Nb, O, and W show a homogeneous distribution, demonstrating the homogenous doping of the W element in the Bi_3_TiNbO_9_‐W nanosheets (Figure 2g; Figure S3, Supporting Information). Inductively coupled plasma atomic emission spectroscopy (ICP‐OES) reveals a W doping content of ≈5% in the Bi_3_TiNbO_9_‐W.

The element composition and chemical states of the Bi_3_TiNbO_9_‐W were characterized using X‐ray photoelectron spectroscopy (XPS). In the Nb 3*d* spectrum of Bi_3_TiNbO_9_‐W, two additional peaks at 209.79 and 207.02 eV are absent in Bi_3_TiNbO_9_, align well with the Nb 3*d* peak positions of Nb_2_O_5_ (Figure 2h,i). The chemical states of Bi 4*f*, Ti 2*p*, and O 1*s* of Bi_3_TiNbO_9_‐W are similar to those in Bi_3_TiNbO_9_ (Figures S4–S6, Supporting Information). These observations suggest that W^6+^ ions occupy the Nb^5+^ sites within the perovskite structure. Density functional theory (DFT) calculations were conducted to investigate the effect of W doping on the electronic structure of Bi_3_TiNbO_9_. Based on the ion valence and radius analysis, W atoms is capable of substituting the Ti and Nb atoms in Bi_3_TiNbO_9_‐W. Accordingly, the formation energies of Bi_3_TiNbO_9_‐W (2 × 2 × 1) were calculated with either one Ti or one Nb atom replaced by W. Therefore, the electronic structure calculations focus on the Nb replacement by W in Bi_3_TiNbO_9_‐W. Compared with the pristine Bi_3_TiNbO_9_ (bandgap of 2.79 eV), Bi_3_TiNbO_9_‐W exhibits smaller bandgap values (2.35–2.50 eV), which are attributed to the lower energy of W 5d orbitals contributing to the CBM, as shown in Figure 2j,k and Figure S7 (Supporting Information). And the doped W acts as a donor, gradually reducing the gap between the Fermi level and CBM from bulk to surface, creating a built‐in electric field along the c‐axis that drives photogenerated electron migration from bulk to surface in Bi_3_TiNbO_9_‐W.

### 2.2 Optical Properties and Photocatalytic Performance of the Ferroelectric Photocatalyst

**Figure 3:**
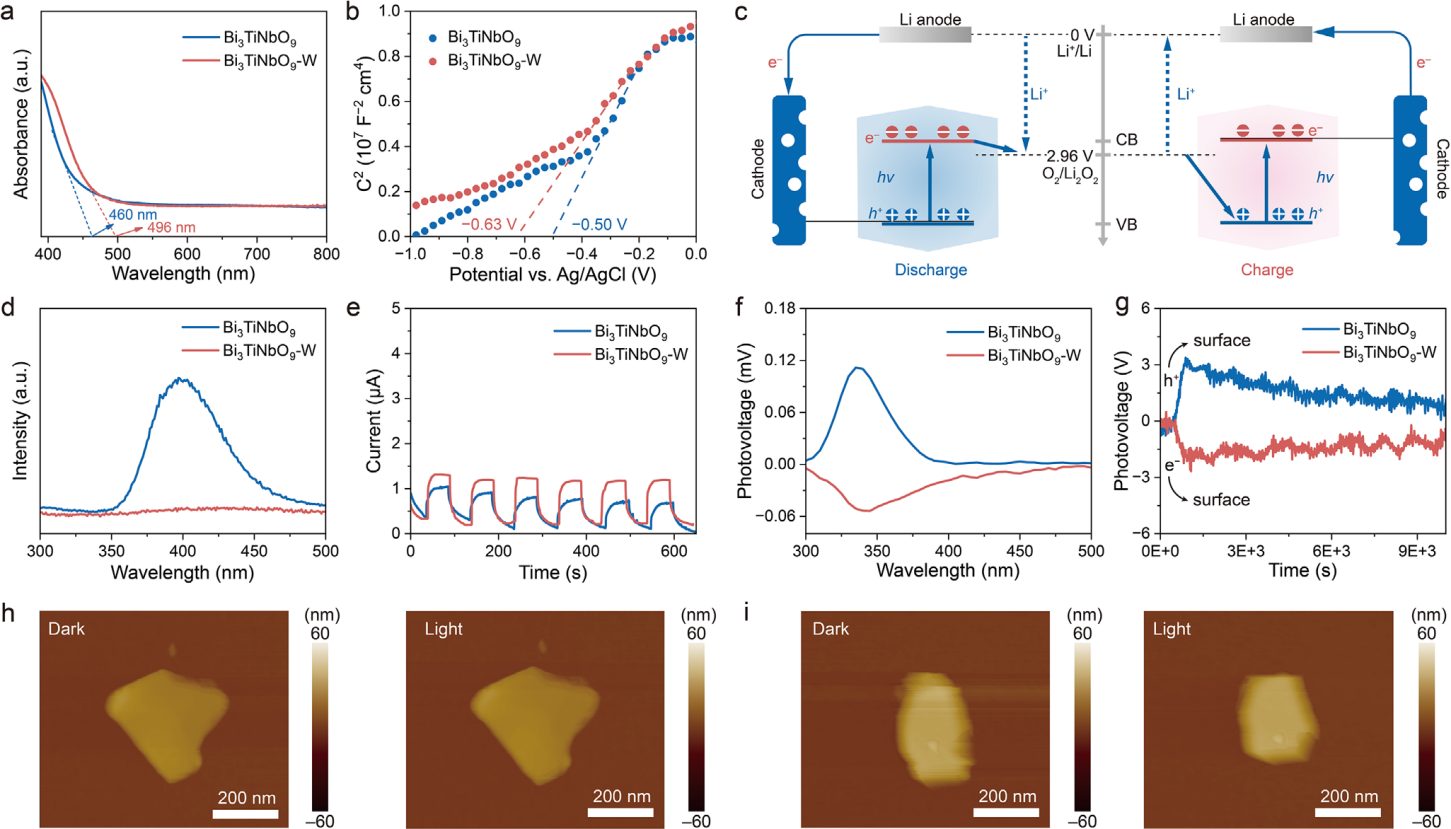
a) UV–vis absorption spectra and b) Mott–Schottky plots of Bi_3_TiNbO_9_ and Bi_3_TiNbO_9_‐W. c) The reaction mechanism of the photo‐assisted Li–O_2_ batteries with ferroelectric photocatalyst cathode. d) PL spectra of Bi_3_TiNbO_9_ and Bi_3_TiNbO_9_‐W. e) Photocurrent response to UV light of Bi_3_TiNbO_9_ and Bi_3_TiNbO_9_‐W. f) SPV spectra and g) TPV response of multiple Bi_3_TiNbO_9_ and Bi_3_TiNbO_9_‐W crystals. AFM height image of h) Bi_3_TiNbO_9_ and i) Bi_3_TiNbO_9_‐W in the dark (left) and light (right).

The optical absorption properties of the Bi_3_TiNbO_9_ and Bi_3_TiNbO_9_‐W were analyzed using ultraviolet‐visible spectroscopy (UV–vis). Compared to Bi_3_TiNbO_9_, Bi_3_TiNbO_9_‐W demonstrated enhanced light harvesting in the visible region, with a notable red shift in the absorption edge from 460 to 496 nm (**Figure** **3**a). As a result, a smaller bandgap (2.50 eV) of Bi_3_TiNbO_9_‐W was calculated than that of Bi_3_TiNbO_9_ (2.70 eV), which lowers the required energy for photoexcitation and boosts the electronic conductivity of the photocathode (Figure S8, Supporting Information). To further investigate the properties of Bi_3_TiNbO_9_ and Bi_3_TiNbO_9_‐W, Mott–Schottky (M‐S) plots were recorded at 1 kHz. Both materials exhibited positive slopes in their plots, confirming their behavior as n‐type semiconductors, with electrons as the predominant charge carriers, which is consistent with previous studies.^[^ ^36^ ^]^ The M‐S plots revealed that the flat‐band position (E_FB_) of Bi_3_TiNbO_9_ and Bi_3_TiNbO_9_‐W were determined to be −0.50 and −0.63 V versus Ag/AgCl, which were further calculated to be 3.15 and 3.02 V versus Li^+^/Li, respectively (Figure 3b). Given that the CB position is ≈0.2 V more negative than the E_FB_, the corresponding CB of Bi_3_TiNbO_9_ and Bi_3_TiNbO_9_‐W were estimated to be 2.95 and 2.82 V. The relative redox potentials for both materials are shown in Figure 3c. The standard potential of O_2_/Li_2_O_2_ lies between the CB and VB of Bi_3_TiNbO_9_ and Bi_3_TiNbO_9_‐W, fulfilling the requirements of photocatalytic ORR/OER. Consequently, the excited photoelectrons and holes can function as the reductants and oxidants in ORR and OER, respectively, favoring the redox processes in Li–O_2_ batteries. Notably, the slope of the Bi_3_TiNbO_9_‐W photocathode was significantly smaller than that of the Bi_3_TiNbO_9_, indicating a higher carrier concentration, which might be ascribed to the shallow donor doping effect originated from the introduction of W^6+^ ions in Bi_3_TiNbO_9_‐W. This higher carrier mobility is capable of extending the lifetime of charge carriers, effectively reducing the electron–hole recombination.

The recombination behavior of photogenerated carriers in the ferroelectric photocatalyst was evaluated through the photoluminescence (PL) spectra (Figure 3d). Compared with Bi_3_TiNbO_9_, the emission peak of the Bi_3_TiNbO_9_‐W showed a lower intensity, suggesting the effectively reduced electron–hole recombination rate by W‐doping. As expected, the Bi_3_TiNbO_9_‐W photocathode, with its enhanced light harvesting ability and reduced recombination rate, exhibited a superior photoresponse compared to the Bi_3_TiNbO_9_ photocathode (Figure 3e). A comprehensive analysis of the photogenerated charge transport behavior between Bi_3_TiNbO_9_ and Bi_3_TiNbO_9_‐W was further conducted. The surface photovoltage (SPV) spectra were used to explore their charge transfer characteristics (Figure 3f). Due to the migration restrictions of the photogenerated electrons along the c‐axis, Bi_3_TiNbO_9_ displayed a positive SPV feature within the optical response range of 300–400 nm. In contrast, the negative signals generated from Bi_3_TiNbO_9_‐W indicate that the altered energy band structure significantly enhance the electron diffusion to the surface. The introduction of W‐doping is prone to generating an additional built‐in electric field, contributing to the enhanced migration of photogenerated electrons from the bulk to the {001} surface. The SPV signal over timescales ranging from microseconds to milliseconds reflected the extended photoresponse time in Bi_3_TiNbO_9_‐W (Figure 3g), which could be attributed to the increased polarization intensity along the a‐axis and the additional electric field along the c‐axis, facilitating the anisotropic migration and more efficient separation of photogenerated charges.

**Figure 4:**
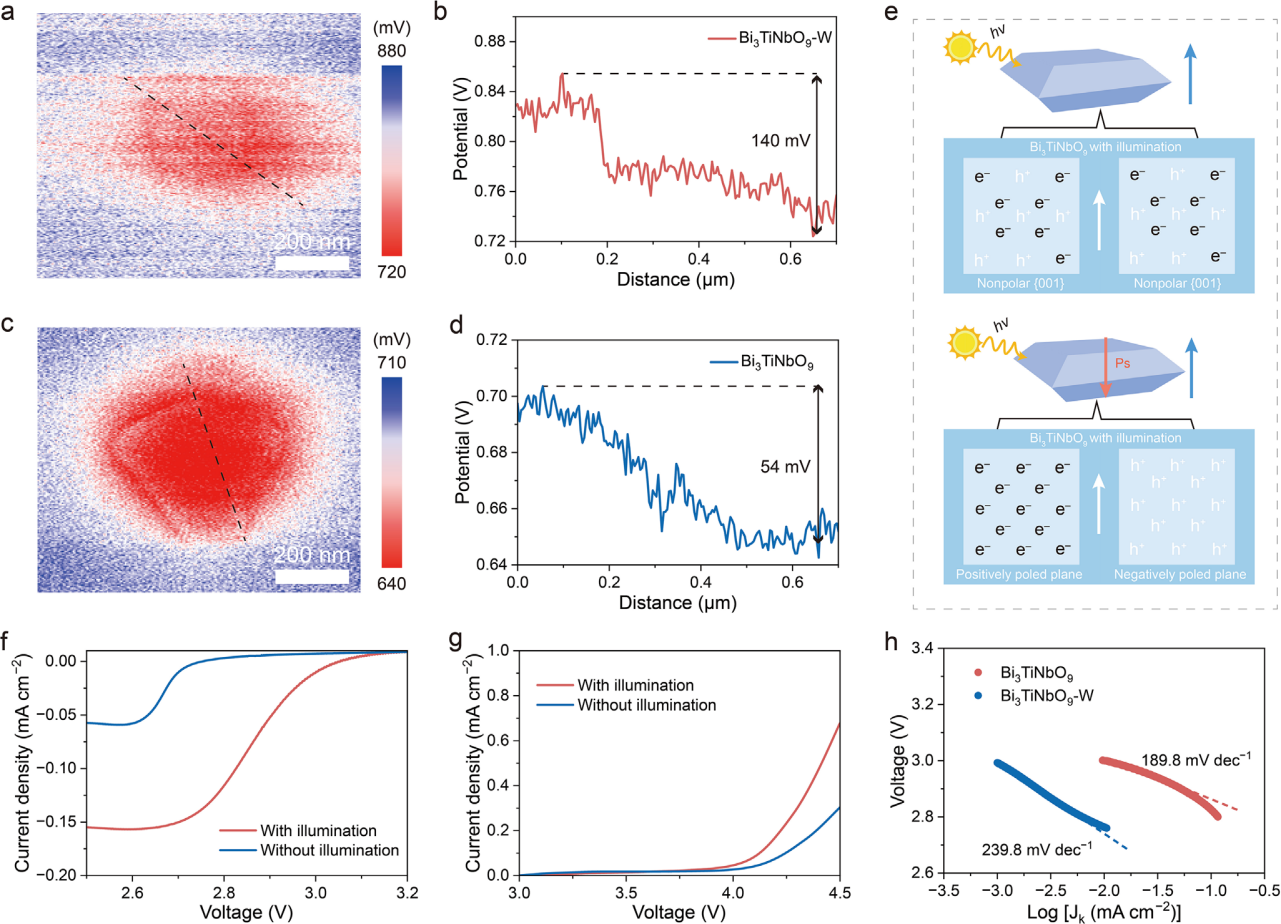
a) SPV image and b) line profiles of the SPV of Bi_3_TiNbO_9_‐W crystal. c) SPV image and d) line profiles of the SPV of Bi_3_TiNbO_9_ crystal. e) Schematic diagram of the photogenerated electron–hole pairs separation process. f) ORR and g) OER polarization curves of Bi_3_TiNbO_9_‐W at 1600 rpm rotating speeds with and without illumination using RRDE. h) The corresponding Tafel plots of Bi_3_TiNbO_9_‐W and Bi_3_TiNbO_9_ with illumination using RRDE during the ORR process.

To further investigate the electron separation and transfer characteristics of Bi_3_TiNbO_9_‐W, Kelvin probe force microscopy (KPFM) was conducted under irradiation. Atomic force microscope (AFM) height mapping images proved the nanosheet morphologies and similar thickness of Bi_3_TiNbO_9_ and Bi_3_TiNbO_9_‐W (Figure 3h,i; Figure S9, Supporting Information). Surface photovoltage (SPV) images highlighted the potential distribution differences between Bi_3_TiNbO_9_ and Bi_3_TiNbO_9_‐W under light and dark conditions. The corresponding SPV curves were derived from the electric potential difference on the material surfaces under these conditions. Compared to Bi_3_TiNbO_9_, a distinct difference was observed in the SPV images and curves, which should be ascribed to the higher electric potential of the Bi_3_TiNbO_9_‐W (**Figure** **4**a,b). Owing to the rapid recombination of photogenerated electron–hole pairs, no significant SPV was detected for Bi_3_TiNbO_9_. In contrast, Bi_3_TiNbO_9_‐W exhibited a pronounced negative SPV, indicating the electron accumulation on the surface of Bi_3_TiNbO_9_‐W. The SPV voltage difference in Bi_3_TiNbO_9_‐W reached ≈140 mV, much higher than that of 54 mV observed for Bi_3_TiNbO_9_ (Figure 4c,d). The SPV signal largely depends on the density of photogenerated carriers, and this substantial difference in SPV indicates that Bi_3_TiNbO_9_‐W can accelerate the carrier separation kinetics and thus effectively extend the lifetime of photoexcited carriers. The mechanism behind the enhanced carrier separation dynamics of Bi_3_TiNbO_9_‐W under illumination is illustrated in Figure 4e. As previously discussed, a built‐in electric field is formed in Bi_3_TiNbO_9_‐W during photoexcitation, promoting the efficient spatial charge separation, which is favorable to achieving the Bi_3_TiNbO_9_‐W‐based photo‐assisted Li–O_2_ batteries by enabling effective light absorption and rapid electron transfer. To assess the photo/electrochemical ORR and OER properties of the ferroelectric photocatalysts, linear scan voltammetry (LSV) curves for Bi_3_TiNbO_9_ and Bi_3_TiNbO_9_‐W were obtained using rotating ring disk electrodes (RRDE), for which Li foil is served as both the counter and reference electrode. During RRDE measurements in 1 m lithium bis(trifluoromethanesulphonyl)imide (LiTFSI)/tretraethylene glycol dimethyl ether (TEGDME), Bi_3_TiNbO_9_‐W displayed a higher onset potential and higher current density under illumination than that without illumination, indicating the beneficial effects of illumination on ORR performance (Figure 4f). For OER, Bi_3_TiNbO_9_‐W also showed significantly improved performance under illumination compared to the non‐illuminated sample and Bi_3_TiNbO_9_ (Figure 4g; Figure S10, Supporting Information), further highlighting the positive influence of illumination on both the ORR and OER activities. Moreover, Bi_3_TiNbO_9_‐W exhibited a lower Tafel slope of 189.8 mV dec^−1^, compared to 239.8 mV dec^−1^ for Bi_3_TiNbO_9_ under illumination, demonstrating superior ORR and OER kinetic activity of Bi_3_TiNbO_9_‐W (Figure 4h).

### 2.3 Adoption of Ferroelectric Photocatalysts in Photo‐Assisted Li–O_2_ Batteries

**Figure 5:**
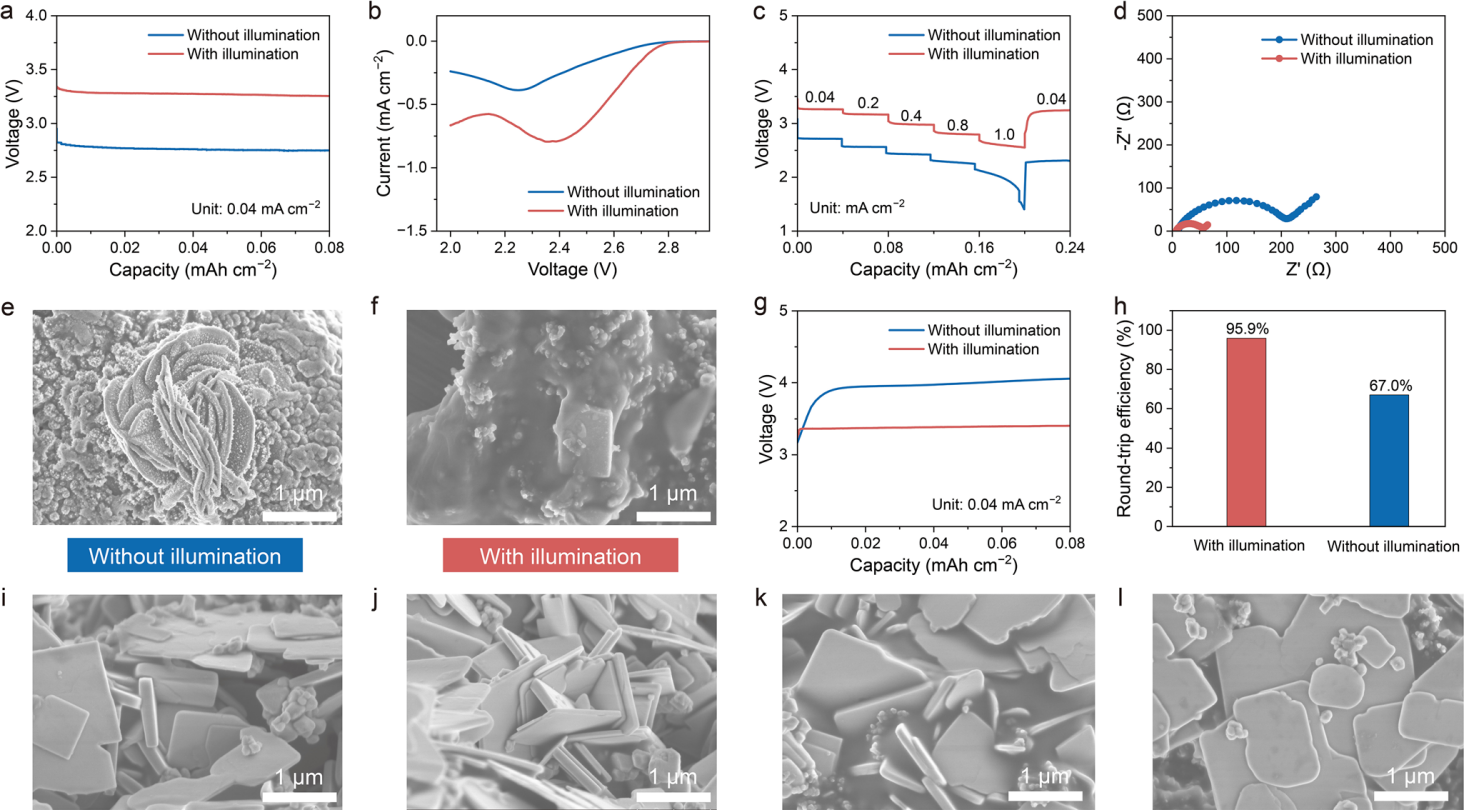
a) The discharge profiles of the Li–O_2_ batteries at 0.04 mA cm^−2^ with and without illumination. b) Cathodic CV curves of the Li–O_2_ batteries with and without illumination at a scan rate of 0.1 mV s^−1^. c) Rate capability of the Li–O_2_ batteries during the discharge process with and without illumination. d) Nyquist plots of the Li–O_2_ batteries with and without illumination. e, f) SEM images of the Bi_3_TiNbO_9_‐W cathodes after being initially discharged without (e) and with (f) illumination. g) The charge profiles of the Li–O_2_ batteries at 0.04 mA cm^−2^ with and without illumination. h) The corresponding comparison of the energy efficiency. i, j) SEM images of the Bi_3_TiNbO_9_‐W cathodes after the 1st charging process without (i) and with (j) illumination. k,l) SEM images of the Bi_3_TiNbO_9_‐W cathodes after the 10th charging process without (k) and with (l) illumination.

During testing, the photo‐assisted Li–O_2_ batteries were assembled using a modified 2025‐type coin cell, with a 500 W Xe lamp as the light source (Figure S11, Supporting Information). The illumination effect on the Li–O_2_ system was investigated separately during both the charging and discharging processes. Under illumination, the Li–O_2_ battery with the Bi_3_TiNbO_9_‐W cathode exhibited a discharge voltage plateau of ≈3.25 V at 0.04 mA cm^−2^, significantly higher than the discharge voltage observed without illumination (2.75 V, as shown in **Figure** **5**a) and the discharge plateau of the Bi_3_TiNbO_9_ cathode, as expected (Figure S12, Supporting Information). The cathodic cyclic voltammetry (CV) curves, obtained at a scan rate of 0.1 mV s^−1^, reflect the results from the galvanostatic discharge plots (Figure 5b). A higher cathodic current density was observed in the CV plot of the photo‐assisted Li–O_2_ battery, demonstrating superior ORR performance driven by photogenerated electrons. For conventional Li–O_2_ batteries, only when the electrons from the anode possess sufficient energy (a more negative potential) to drive the reduction reaction of O_2_ to Li_2_O_2_. Consequently, the discharge voltage is typically lower than the O_2_/Li_2_O_2_ redox couple equilibrium voltage (2.96 V vs Li^+^/Li). In contrast, in the novel photo‐assisted Li–O_2_ battery systems, electrons are photoexcited to the CB under illumination, reaching a potential more negative than 2.96 V versus Li^+^/Li, thereby significantly enhancing the ORR kinetics. Simultaneously, electrons generated by the oxidation of the Li anode are injected into the VB of the cathode, resulting in a theoretical increase (ΔE_dis_) in the overall operating voltage of the battery, exceeding the equilibrium voltage, as depicted in Figure 3c. Despite the battery showed a significant difference in the discharge voltage with and without illumination, in situ differential electrochemical mass spectrometry (DEMS) demonstrated that both the discharge process were dominated by O_2_ consumption (Figure S13, Supporting Information).

In addition to the energy provided by the photoexcited electrons to assist the ORR process, the rapid photoexcitation response and enhanced mass transfer under illumination also favor the electrochemical reactions by reducing the battery polarization. This effect becomes more pronounced at higher rates, as demonstrated in Figure 5c. Even at a high current density of 1 mA cm^−2^, the photo‐assisted Li–O_2_ batteries maintained a discharge voltage of ≈2.55 V, significantly outperforming the non‐illuminated battery, which dropped below 2 V at the same current density. To further explore the kinetic properties of the batteries with and without illumination, electrochemical impedance spectra (EIS) were examined. Nyquist plots, the semicircle in the high‐to‐medium frequency region represents the charge transfer resistance (R_ct_), which arises from the resistance at the electrode/electrolyte interfaces. As shown in Figure 5d, the R_ct_ for the battery was much lower than that of the non‐illuminated cell, indicating superior interfacial charge transfer kinetics in the photo‐assisted Li–O_2_ battery. Moreover, the unique discharge mechanism and fast electron conductivity under illumination would influence the deposition and morphology of the discharge products. Without illumination, the discharge products were randomly stacked on the nanosheet‐structured cathode, forming nanosheet‐stacked structures with several hundred nanometers in size (Figure 5e), which would deposit on the electrode surface, impeding the contact between the electrolyte and active sites and destroy the nanostructure of the cathode, severely limiting the following electrochemical reactions. In contrast, the discharge products were uniformly distributed as thin films on the Bi_3_TiNbO_9_‐W cathode (Figure 5f). This uniform deposition is favorable to preserving the active sites after the discharge process, and thus enhancing the electrochemical kinetics during the subsequent charging process.

The photo‐electrochemical performance of the Li–O_2_ battery with Bi_3_TiNbO_9_‐W cathode during charging was further explored under illumination. As shown in Figure 5g, benefiting from the photo‐assistance, the charge voltage significantly dropped from 4.10 to 3.39 V under illumination, which effectively suppressed the polarization of the Bi_3_TiNbO_9_‐W cathode. This reduction aligns with the LSV plots for the OER process (Figure S14, Supporting Information). Predictably, the charge plateau of the Bi_3_TiNbO_9_‐W cathode was also lower than that of the pristine Bi_3_TiNbO_9_ cathode under illumination (Figure S15a, Supporting Information). The small overpotential of 0.14 V under illumination led to an impressive round‐trip efficiency of 95.9%, primarily driven by the photoenergy contribution (Figure 5h). However, the Li–O_2_ battery with the Bi_3_TiNbO_9_ cathode under illumination showed a discharge voltage of 2.96 V and a charge voltage of 3.53 V with a round‐trip efficiency of 83.9%, which was much lower than the battery with Bi_3_TiNbO_9_‐W cathode under illumination (Figure S15b, Supporting Information). The superior capability of Bi_3_TiNbO_9_‐W cathode toward catalytic oxidation of Li_2_O_2_ can be further confirmed by the full discharge/charge curves of Li–O_2_ batteries with/without illumination (Figure S16, Supporting Information). As a result, the photo‐assisted charging process not only reduced the electricity consumption compared with the conventional Li–O_2_ batteries but also improved the decomposition kinetics of the discharge products. SEM images of the recharged Bi_3_TiNbO_9_‐W cathodes after different cycling numbers were captured to illustrate the OER process under both illuminated and non‐illuminated conditions (Figure 5i–l). After the initial recharge, most of the discharge product, Li_2_O_2_, was decomposed, regardless of whether the illumination was applied (Figure 5i,j). However, after 10 cycles without illumination, Li_2_O_2_ accumulated on the surface of the Bi_3_TiNbO_9_‐W cathode (Figure 5k; Figure S17, Supporting Information), in sharp contrast to the Bi_3_TiNbO_9_‐W cathode cycled under illumination, where no such accumulation was observed (Figure 5l; Figure S18, Supporting Information).

**Figure 6:**
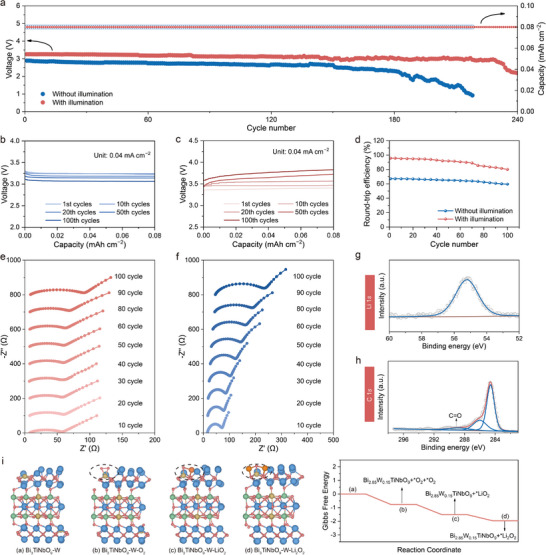
a) Cycling performance of the Li–O_2_ batteries at 0.04 mA cm^−2^ with and without illumination. The discharge b) and the charge c) curves of the Li–O_2_ batteries at 0.04 mA cm^−2^ with illumination for 100 cycles. d) The round‐trip efficiency of the Li–O_2_ batteries at a current density of 0.04 mA cm^−2^ with and without illumination. In situ EIS plots of the Li–O_2_ batteries e) with and f) without illumination during cycling. g) Li 1*s* and h) C 1*s* XPS spectra of the Bi_3_TiNbO_9_‐W cathode after being discharged with illumination. i) Optimized geometries of Bi_3_TiNbO_9_‐W, Bi_3_TiNbO_9_‐W‐O_2_, Bi_3_TiNbO_9_‐W‐LiO_2_, Bi_3_TiNbO_9_‐W‐Li_2_O_2_, respectively and the calculated free‐energy diagrams for Li_2_O_2_ generation with illumination.

The cycling stability of the photo‐assisted Li–O_2_ battery was assessed through the galvanostatic charge‐discharge measurements at a current density of 0.04 mA cm^−2^ (**Figure** **6**a). The discharge terminal voltage of the illuminated battery consistently remained higher than that of the battery without illumination (up to 240 cycles, 960 h), demonstrating the sustained performance of the Bi_3_TiNbO_9_‐W photocatalyst used in the Li–O_2_ battery. As shown in Figure 6b, the discharge voltage plateau gradually decreased to 3.10 V after 100 cycles, indicating the excellent stability of the ORR process under illumination. Furthermore, the charge voltage slightly rose to 3.80 V after 100 cycles under illumination (Figure 6c). The increased polarization observed in the photo‐assisted electrochemical processes after extended cycling might be attributed to the evaporation and decomposition of the electrolyte, a phenomenon similar to that observed in the non‐illuminated batteries (Figure S19, Supporting Information). As confirmed by ex‐situ XRD patterns of the Bi_3_TiNbO_9_‐W cathodes (Figure S20, Supporting Information), the photocatalyst remained stable, while only mild electrolyte decomposition occurred after cycling with or without illumination, as indicated by the NMR spectra (Figure S21, Supporting Information). Moreover, no obvious side product was accumulated on the Bi_3_TiNbO_9_‐W cathodes cycled with illumination (Figure S22, Supporting Information). Despite slight interactions between the electrolyte and electrode in the photo‐assisted Li–O_2_ battery, the round‐trip efficiency was dramatically improved, maintaining ≈80% after 100 cycles (Figure 6d), which is largely attributed to the lower charge voltage and higher discharge voltage resulting from the illumination. Compared with the reported representative photo‐assisted Li–O_2_ battery, the Bi_3_TiNbO_9_‐W cathode exhibits superiority in cycling life of 240 cycles and a higher initial round‐trip efficiency of 95.9% (Table S1, Supporting Information).

In situ EIS tests were conducted at different stages during long‐term cycling to evaluate the reversibility of the photo‐assisted Li–O_2_ battery. As shown in Figure 6e, the impedance of the photo‐assisted batteries increases slowly during cycling, suggesting better electronic conductivity of the discharge products and fewer accumulated side products. In sharp contrast, the impedance of the batteries without illumination increases rapidly, ascribed to the accumulation of the insulting discharge products and side products (Figure 6f). Ex situ XPS analysis was carried out on the cathodes after full discharge and recharge both with and without illumination. As shown in Figure 6g,h, peak changes of the Li 1s and C 1s in the XPS spectra indicate the Li_2_O_2_ formation on the illuminated cathode, with no observable peaks corresponding to byproducts. However, for the battery without illumination, the Li 1s region of the discharged cathode shows the presence of Li_2_CO_3_ peaks at 55.8 eV (Figure S23, Supporting Information), indicating the existence of the insulated byproducts, which primarily originated from the electrolyte decomposition at high charge potentials, leading to the increased battery impedance, also consistent with the EIS results. Density functional theory (DFT) calculations were employed to investigate the superior photo/electrocatalytic performance of Bi_3_TiNbO_9_‐W by calculating the generated Li_2_O_2_ during discharge on the surface of Bi_3_TiNbO_9_‐W and Bi_3_TiNbO_9_, with optimized structures. For the ORR process, O_2_ is reduced to Li_2_O_2_ on the illuminated Bi_3_TiNbO_9_‐W cathode via the intermediates of Bi_3_TiNbO_9_‐W‐O_2_ and Bi_3_TiNbO_9_‐W‐LiO_2_ (Figure 6i). Such a reaction route is thermodynamically favorable with the downhill free energy, taking advantage of the additional contribution of solar energy.

## 3 Conclusion

In summary, an efficient ferroelectric photocatalyst Bi_3_TiNbO_9_‐W with spontaneous polarization‐induced internal electric fields was developed as the photocathode for photo‐assisted Li–O_2_ batteries. Taking advantage of the introduction of the W dopant, an additional built‐in electric field was formed from the surface to the bulk to inhibit the recombination of the photogenerated electrons and holes, enabling the accelerated reaction kinetics on Bi_3_TiNbO_9_‐W. The generated photoelectrons and holes on Bi_3_TiNbO_9_‐W with strong redox capabilities extended the visible‐light adsorption, and further prolonged the lifespan on Bi_3_TiNbO_9_‐W. As a result, the discharge voltage of the photo‐assisted Li–O_2_ batteries with Bi_3_TiNbO_9_‐W is significantly raised to 3.25 V and the charge voltage is lowered to 3.39 V at 0.04 mA cm^−2^, delivering an ultrahigh energy conversion efficiency of 95.9%. Moreover, the photo‐assisted Li–O_2_ batteries with Bi_3_TiNbO_9_‐W photocathode show a superior rate capability and stable cycling stability up to 960 h. This investigation highlights the ferroelectric photocatalyst for promoted cathode reaction kinetics in Li–O_2_ batteries, providing an inspiring thought on the rational design of other advanced photo‐assisted battery systems.

## Conflict of Interest

The authors declare no conflict of interest.

## Supporting information



Supporting Information

## Data Availability

The data that support the findings of this study are available in the supplementary material of this article.
