# Supplementary material for: Tunable High‐Performance Photo‐Assisted Li–O2 Batteries by the Construction of Ferroelectric Photocathode
Source: Adv Sci (Weinh). 2025 May 5;12(21):2414616. doi: 10.1002/advs.202414616 (PMC12140290; doi:10.1002/advs.202414616)
Supplement: Supplementary file 1 — Supporting Information [file ADVS-12-2414616-s001.docx]

Supporting Information

**Tunable High-performance Photo-assisted Li–O_2_ Batteries by the Construction of Ferroelectric Photocathode**

*Huan-Feng Wang, Yu-Fei Wang*, De-Hui Guan, Xiao-Xue Wang, Xin-Yue Ma, Xin-Yuan Yuan, and Ji-Jing Xu**

Dr. H. Wang, Prof. Y. Wang

College of Food and Chemical Engineering, Zhengzhou Key Laboratory of Functional Electrocatalysis and Chemical Energy Storage, Zhengzhou University of Technology, Zhengzhou, 450044 P. R. China

Dr. D. Guan, Dr. X. Wang, X. Ma, X. Yuan, Prof. J. J. Xu

State Key Laboratory of Inorganic Synthesis and Preparative Chemistry, College of Chemistry, Jilin University, Changchun 130012 P.R. China

Dr. D. Guan, Dr. X. Wang, Prof. J. J. Xu

International Center of Future Science, Jilin University, Changchun 130012 P.R. China

*Correspondence to: 20051038@zzut.edu.cn; jijingxu@jlu.edu.cn

**Experimental Section**

**1. Chemicals and Materials**

All reagents and solvents were commercially available and used as received. Bismuth oxide (Bi_2_O_3_), anatase titanium dioxide (TiO_2_), niobium pentoxide (Nb_2_O_5_), sodium chloride (NaCl), potassium chloride (KCl), tungsten trioxide (WO_3_), polyvinylidene fluoride (PVDF), tretraethylene glycol dimethyl ether (TEGDME), and lithium bis(trifluoromethanesulphonyl)imide (LiTFSI) were purchased from Aladdin Reagent. Deuterium oxide (D_2_O) was purchased from Qingdao Tenglong Weibo Technology co., Ltd. Carbon paper (CP) was purchased from CeTech Co., Ltd.

**2. Materials Preparation**

**Synthesis of Bi_3_TiNbO_9_.** Bi_3_TiNbO_9_ photocatalyst was obtained via a typical solid-state flux method.^[1]^ Bi_2_O_3_ (0.699 g), anatase TiO_2_ (0.08 g), Nb_2_O_5_ (0.133 g), and NaCl-KCl (8 g, a molar ratio of 1:1) were mixed in an agate mortar and ground for 30 min, and then transferred into an alumina crucible, calcined at 800 °C for 2 h. The obtained product was washed with hot deionized water to completely remove the flux reagents, dried in the air, and denoted as Bi_3_TiNbO_9_.

**Synthesis of Bi_3_TiNbO_9_-W.** Bi_3_TiNbO_9_-W photocatalyst was fabricated using Bi_3_TiNbO_9_ electrode as the substrate. Bi_2_O_3_ (0.699 g), anatase TiO_2_ (0.08 g), Nb_2_O_5_ (0.133 g), NaCl-KCl (8 g, a molar ratio of 1:1), and an appropriate amount of WO_3_ were mixed in an agate mortar for 30 min, and then transferred into an alumina crucible, calcined at 800 °C for 2 h. The obtained product was washed with hot deionized water to completely remove the flux reagents, then dried in the air, and denoted as Bi_3_TiNbO_9_-W.

**3. Materials Characterization**

Scanning electron microscopy (SEM, JEOL JSM-6700F) and transmission electron microscopy (TEM, JEM-2200FS electron microscope) were conducted to investigate the microstructures and the morphologies of the samples. X-ray diffraction (XRD) patterns were recorded on a Rigaku D-Max 2550 diffractometer using Cu Kα radiation. Raman spectra were collected on a LabRAM HR Evolution with a 532 nm laser as the excitation source under ambient conditions. X-ray photoelectron spectroscopy (XPS) was recorded on an ESCALAB 250 spectrometer. An ultraviolet-visible spectrophotometer (UV-vis, PerkinElmer Lambda 950) was applied to characterize the absorption spectra of the samples. A photoluminescence emission spectrometer (HORIBA FluoroMax-4) was utilized to evaluate the photoluminescence emission spectra. ^1^H Nuclear Magnetic Resonance (NMR) spectra were collected on a Bruker Avance II 400 spectrometer using D_2_O for dissolution. AFM measurements were performed by a commercial AFM setup (NT-AIST, HORIBA, Japan) in the force mapping mode using a gold-coated tip (MikroMasch, USA).

**4. Photoelectrochemical and Electrochemical Measurement**

The as-prepared integrated cathodes were cut into small wafers with a diameter of 10 mm. A 500 W Xe lamp served as the light source, and the power was fixed at 100 mW cm^−2^.

**Mott-Schottky (M-S) plots of the photocathode.** M-S plots of the integrated cathodes were created by employing a three-electrode system in 0.5 M NaSO_4_ solution (pH=7.0), with a Pt foil counter-electrode and Ag/AgCl reference electrode. The potentials were converted to RHE according to the equation (1-2):

$\text{V}_{\text{vs.RHE}}\text{=}\text{V}_{\text{vs.Ag/AgCl}}\text{+0.199+0.059×pH}$ (1)

$\text{V}_{\text{vs.Li+/Li}}\text{=}\text{V}_{\text{vs.RHE}}\text{+3.04 V}$ (2)

Commonly, the carrier density was calculated based on the Mott-Schottky equation (3):

$N_{d}=(\frac{2}{e_{0}\varepsilon_{0}\varepsilon})[\frac{d\left( \frac{1}{C^{2}} \right)}{dV}]$ (3)

where *N*_d_ is the donor density, *e*_0_ indicates the electron charge (1.602×10^−19^ C), ε_0_ represents the vacuum permittivity (8.854×10^−12^ F m^−1^), ε is the dielectric constant of the material, and d(1/C^2^)/dV equals to the slope of the M-S plots. Therefore, the donor density is inversely proportional to the slope of the M-S plots. The flat-band potential (*V*_fb_) was obtained by extrapolating the x-intercept of the corresponding line.^[2]^

**KPFM measurements.** The spring constant of the cantilever was 5 N m^−1^ and a resonance frequency of 160 kHz. The tips were cleaned with the Ar ion bombardment to remove the contamination. The nano-materials were dispersed on the silicon wafer and dried naturally. The light coupled from an LED light source (LFM375) by an optical fiber (SMA-905) illuminates the nano-materials at the incident angle of 45 degrees. The topography and KPFM (Kelvin probe force microscopy) images were recorded in a closed chamber to lower the noise.

**Rotating ring disk electrode (RRDE) measurements.** All electrochemical measurements of ORR were performed with a CS2350H Electrochemical Analyzer and a Pine Modulated Speed Rotator. In particular, the rotating disk or ring-disk electrode (RDE electrode surface: 0.1966 cm^2^, RRDE electrode surface: 0.2475 cm^2^) was selected as the working electrode, Li foil both as the counter electrode and the reference electrode. About 15 µL of the catalyst ink (5 mg active materials, 1 mg Super P conductive carbon, 300 µL ethanol, 150 µL isopropanol, and 40 µL Nafion solution (5 wt%, DuPont) was coated onto the working electrode.

**Assembling and testing of the Li–O_2_ batteries*.*** All Li–O_2_ batteries were assembled in an Ar-filled glovebox. The electrolyte was prepared by dissolving 1 M LiTFSI in TEGDME. Glass fiber and Li foil was used as the separator and the counter electrode, respectively, in a 2025-type coin cell with a window for illumination and O_2_ diffusion. The assembled cell was enclosed in a quartz chamber filled with O_2_, and tested on a LAND CT2001A multichannel battery testing system (Wuhan, China). Linear sweep voltammetry (LSV) and electrochemical impedance spectra (EIS) were performed on a CHI660E electrochemical workstation (Shanghai, China).

**5.** **DFT calculations.** All geometric structures were optimized using the CP2k 2023.1 program package. The DZVP-MOLOPT-SR-GTH basis set and PBE functional in combination with Grimme’s dispersion correction (D3) and Becke−Johnson damping factor (BJ) were used during the geometry optimization and BSSE. In contrast, the HSE06-D3(BJ) was used to calculate the states density. All analyses were carried out using the Multiwfn software package and visualized by VESTA. The crystal lattice was fully relaxed until the atomic force was less than 0.02eV/Angstrom. The energy convergence criterion for self-consistent electronic calculation was set to 10^−6^ eV.

**
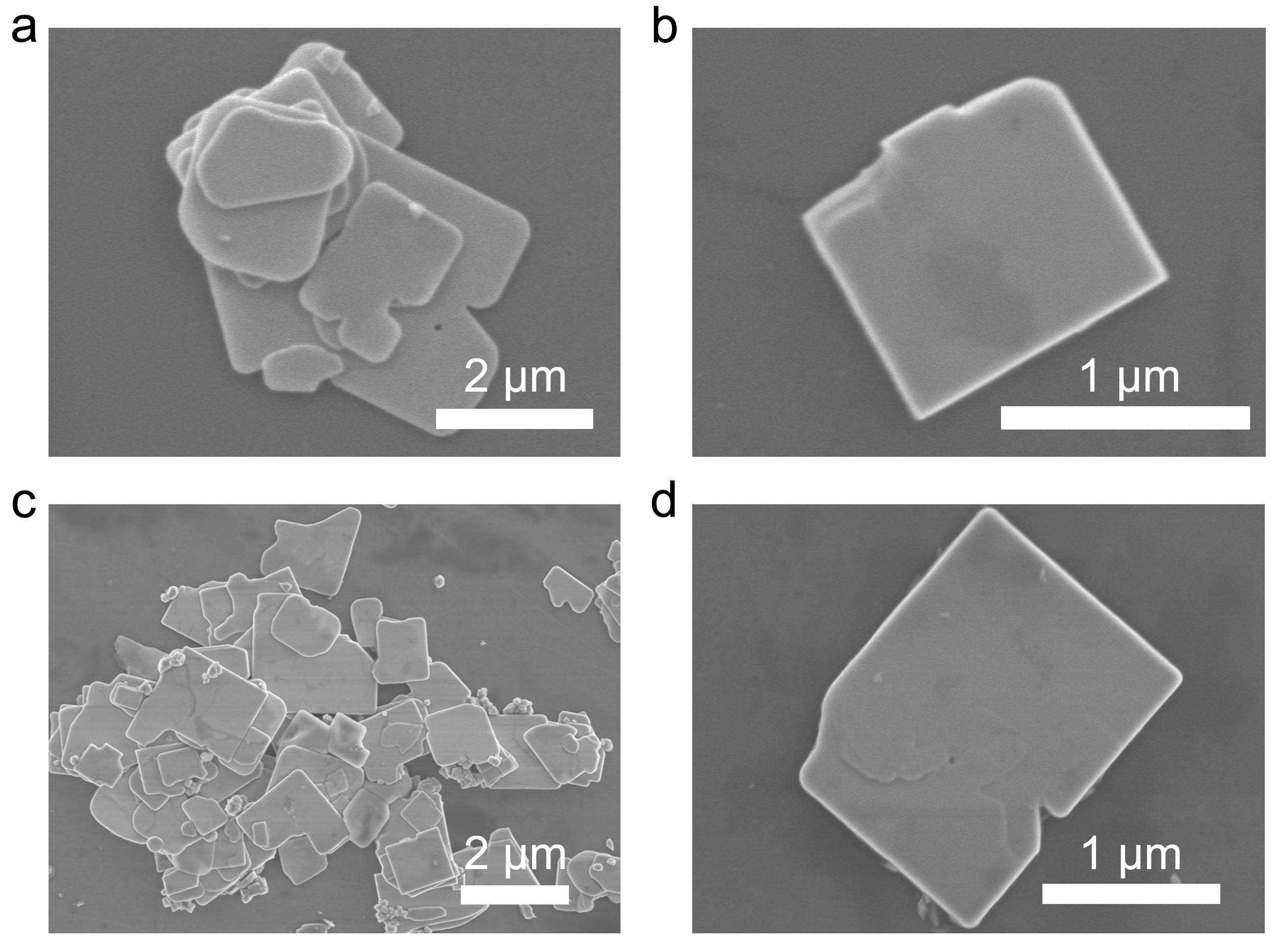
**

**Figure S1.** SEM images of (a-b) Bi_3_TiNbO_9_ and (c-d) Bi_3_TiNbO_9_-W at different magnifications.

**
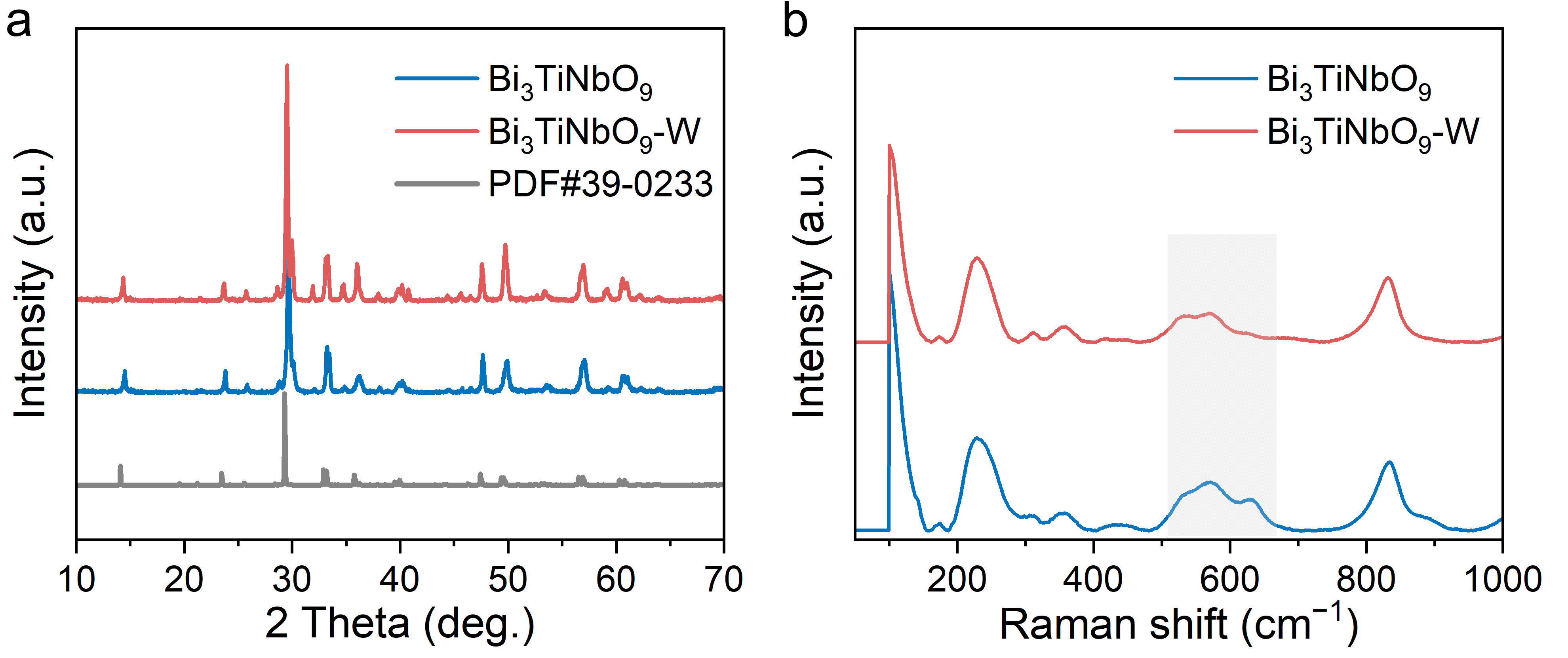
**

**Figure S2.** (a) XRD patterns and (b) Raman spectra of Bi_3_TiNbO_9_ and Bi_3_TiNbO_9_-W.

**
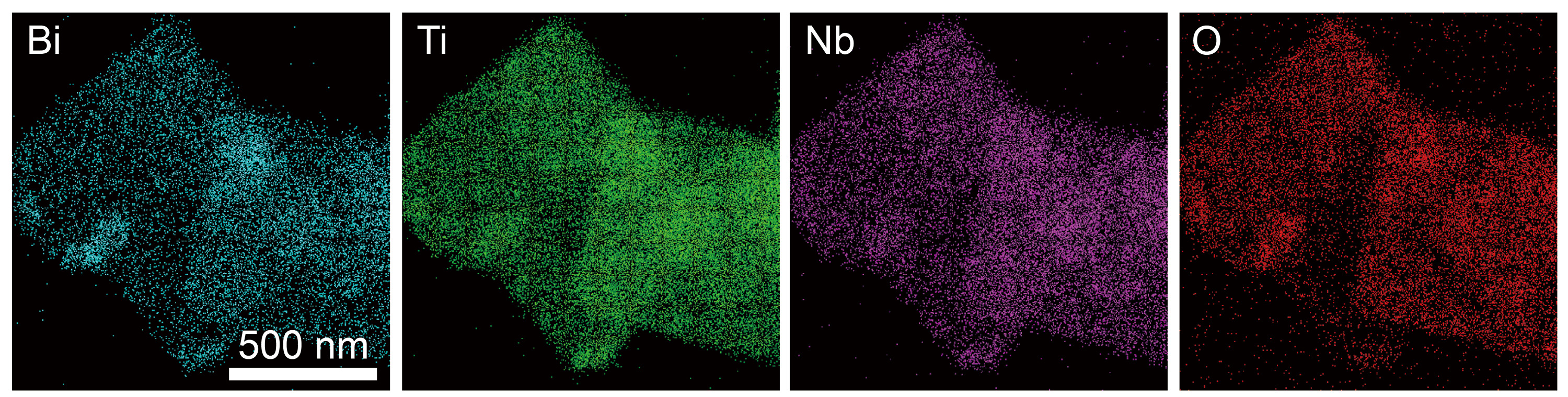
**

**Figure S3.** EDS elemental mapping images of Bi_3_TiNbO_9_.

**
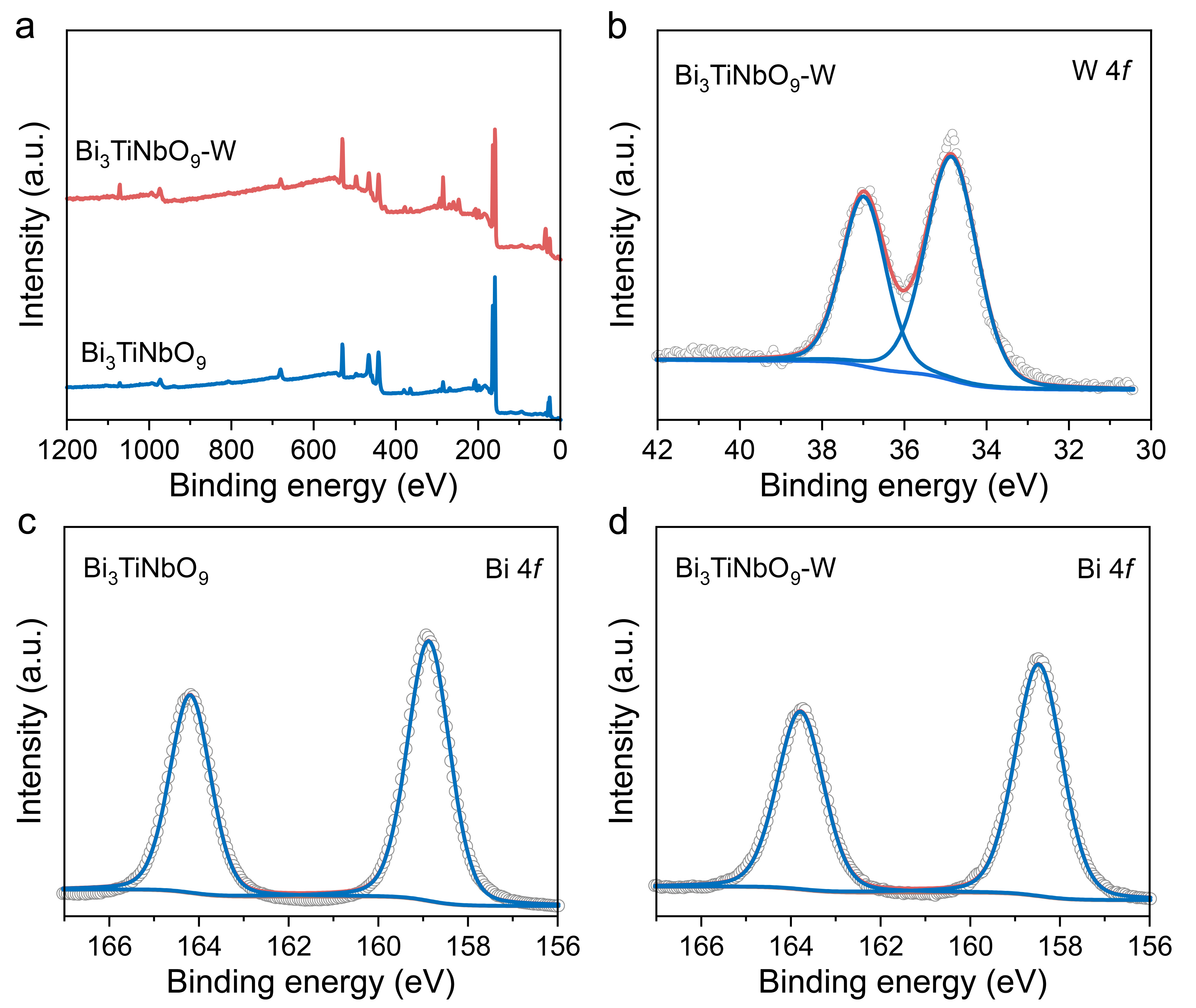
**

**Figure S4.** (a) Survey spectra of Bi_3_TiNbO_9_ and Bi_3_TiNbO_9_-W. (b) W 4*f* high-resolution XPS spectra of Bi_3_TiNbO_9_. Binding energy comparison of Bi 4*f* in (c) Bi_3_TiNbO_9_ and (d) Bi_3_TiNbO_9_-W.

**
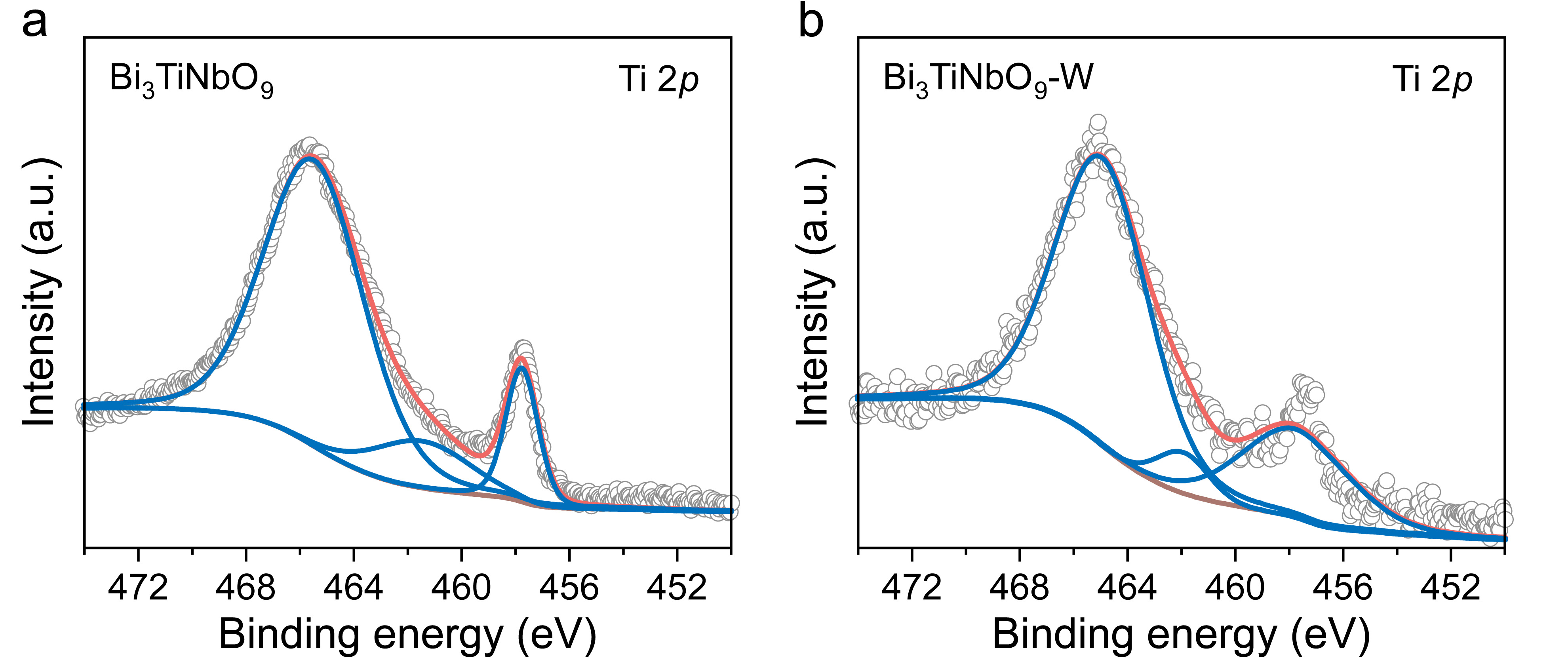
**

**Figure S5.** Binding energy comparison of Ti 2*p* in (a) Bi_3_TiNbO_9_ and (b) Bi_3_TiNbO_9_-W.

**
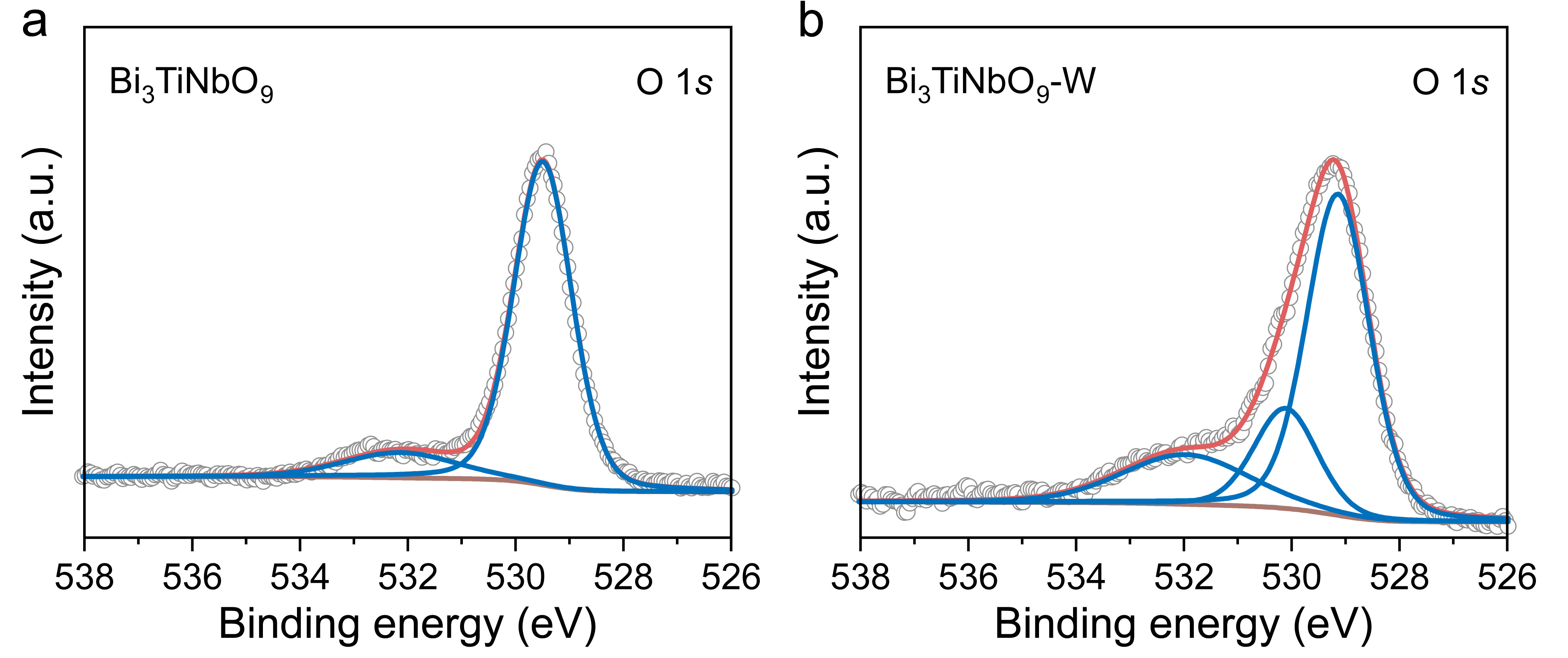
**

**Figure S6.** Binding energy comparison of O 1*s* in (a) Bi_3_TiNbO_9_ and (b) Bi_3_TiNbO_9_-W.

**
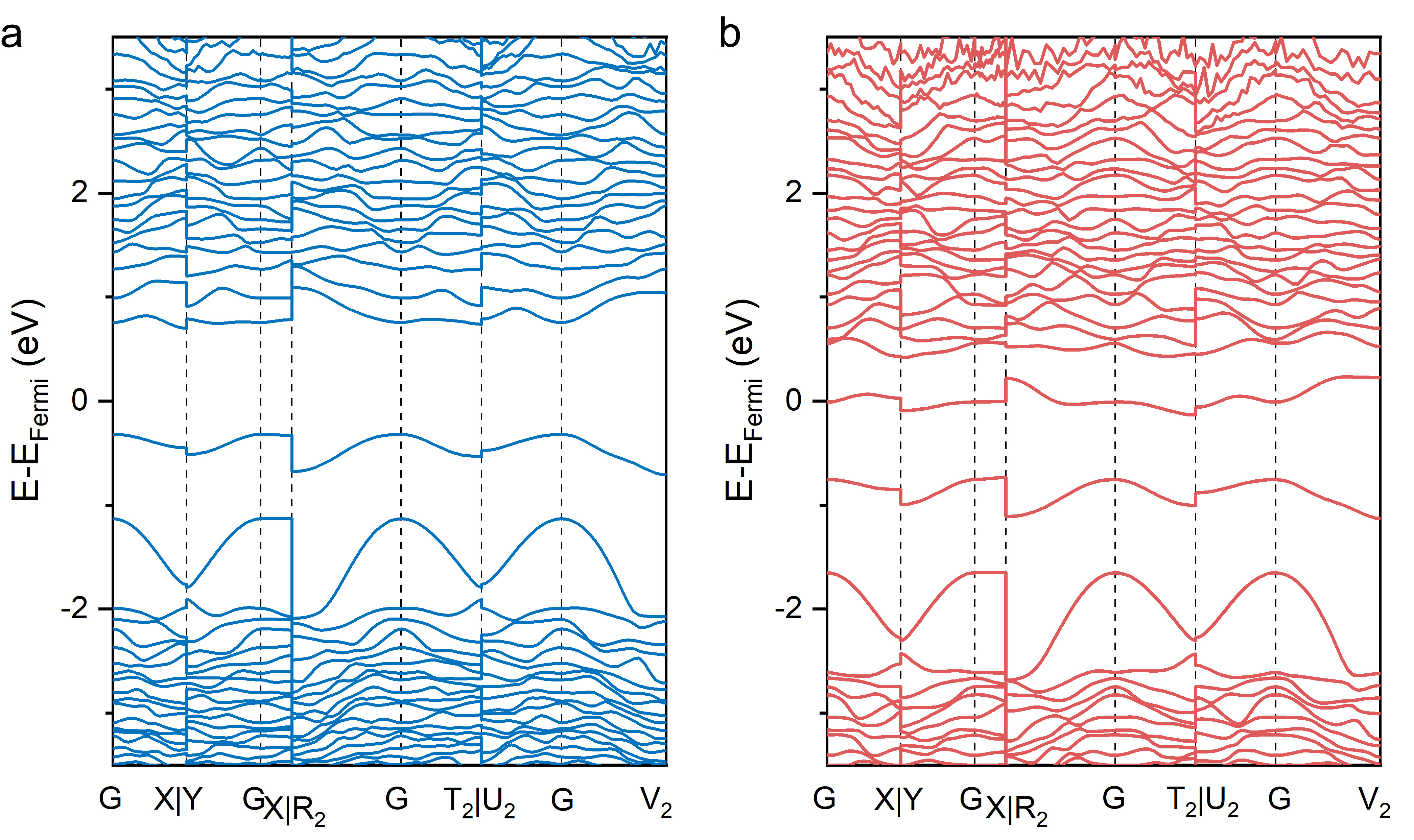
**

**Figure S7.** The calculated band structures of (a) Bi_3_TiNbO_9_ and (b) Bi_3_TiNbO_9_-W.

**
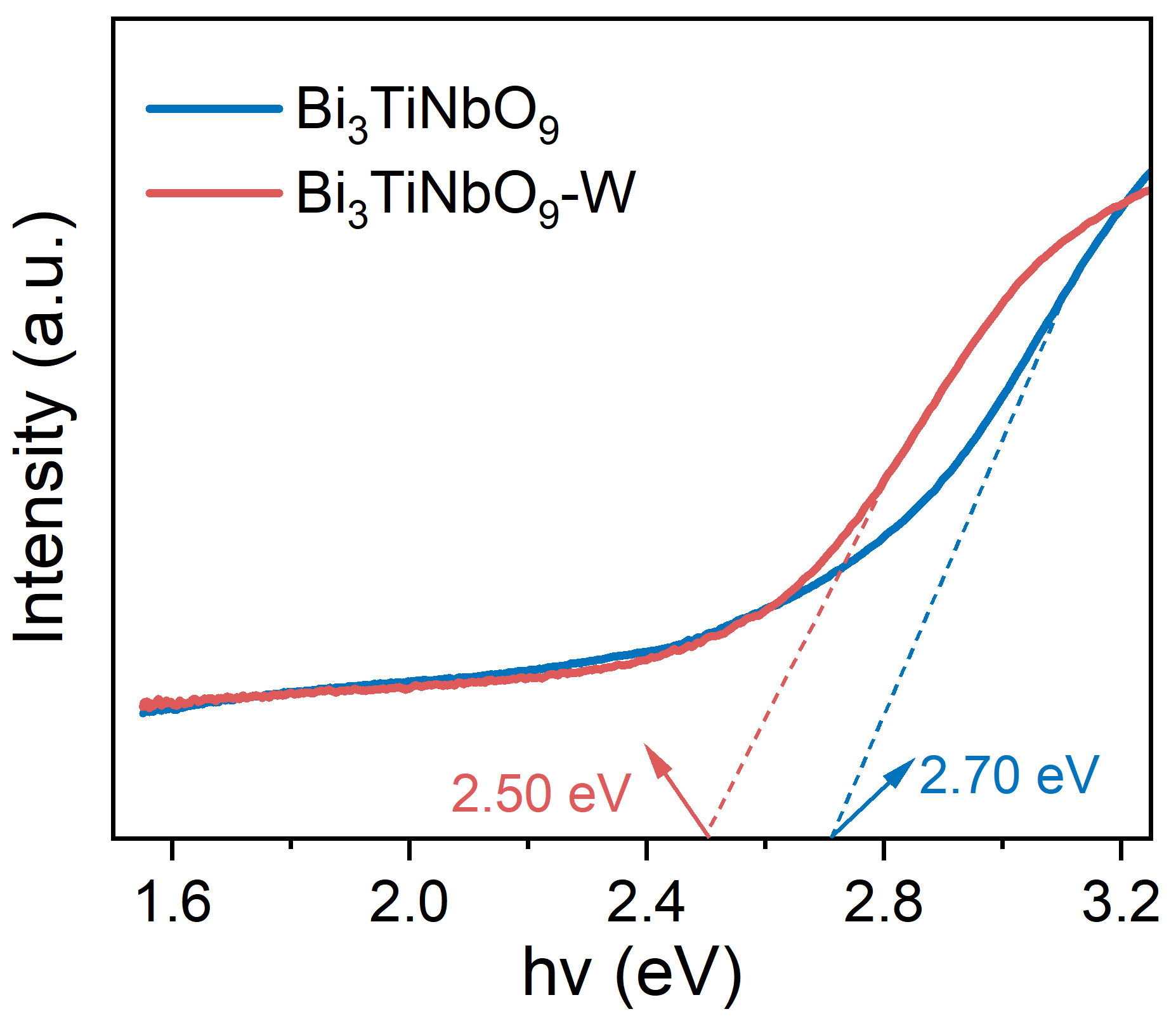
**

**Figure S8.** Tauc plots for the bandgap calculation of Bi_3_TiNbO_9_ and Bi_3_TiNbO_9_-W.


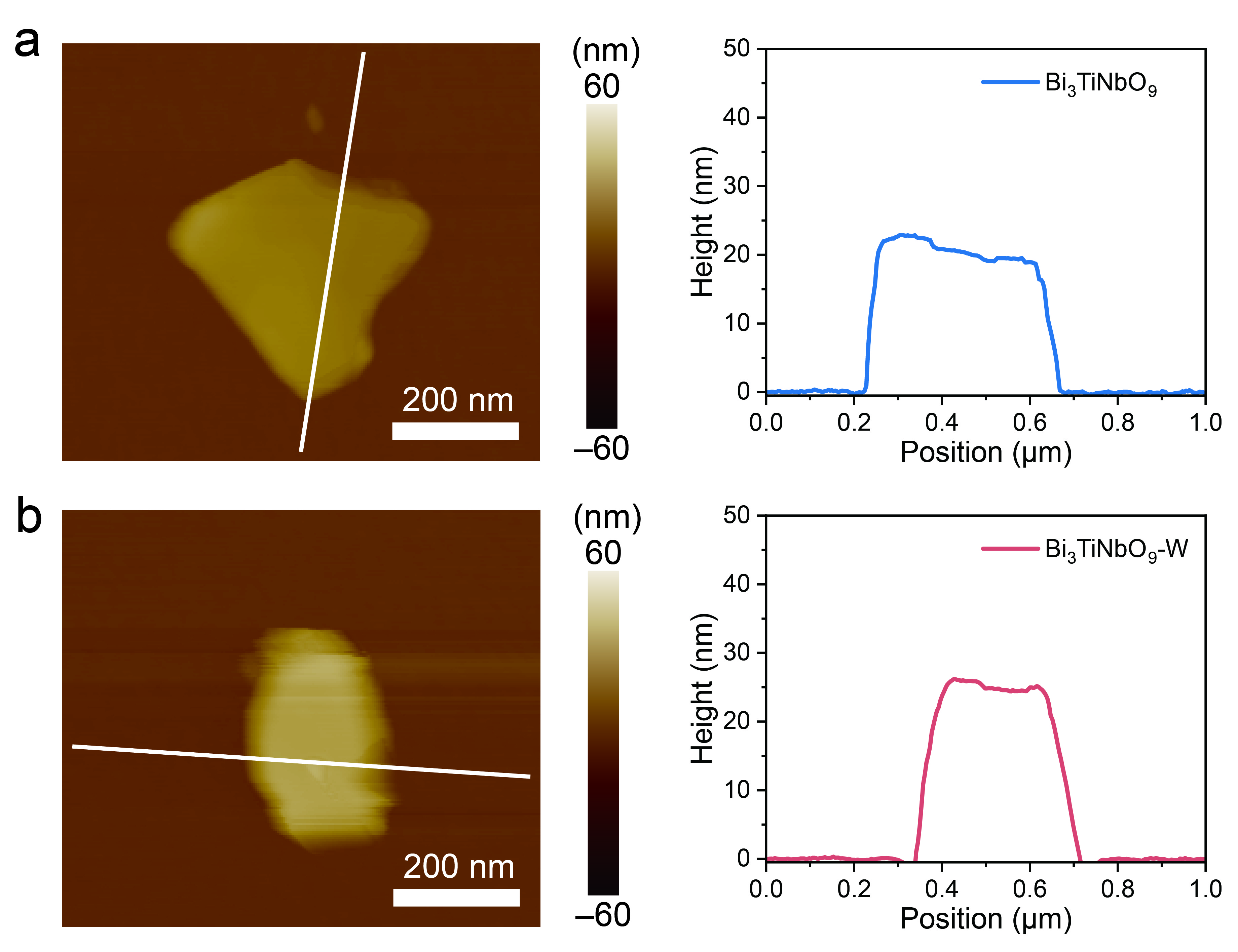


**Figure S9.** AFM height image and corresponding height profile of (a) Bi_3_TiNbO_9_ and (b) Bi_3_TiNbO_9_-W.


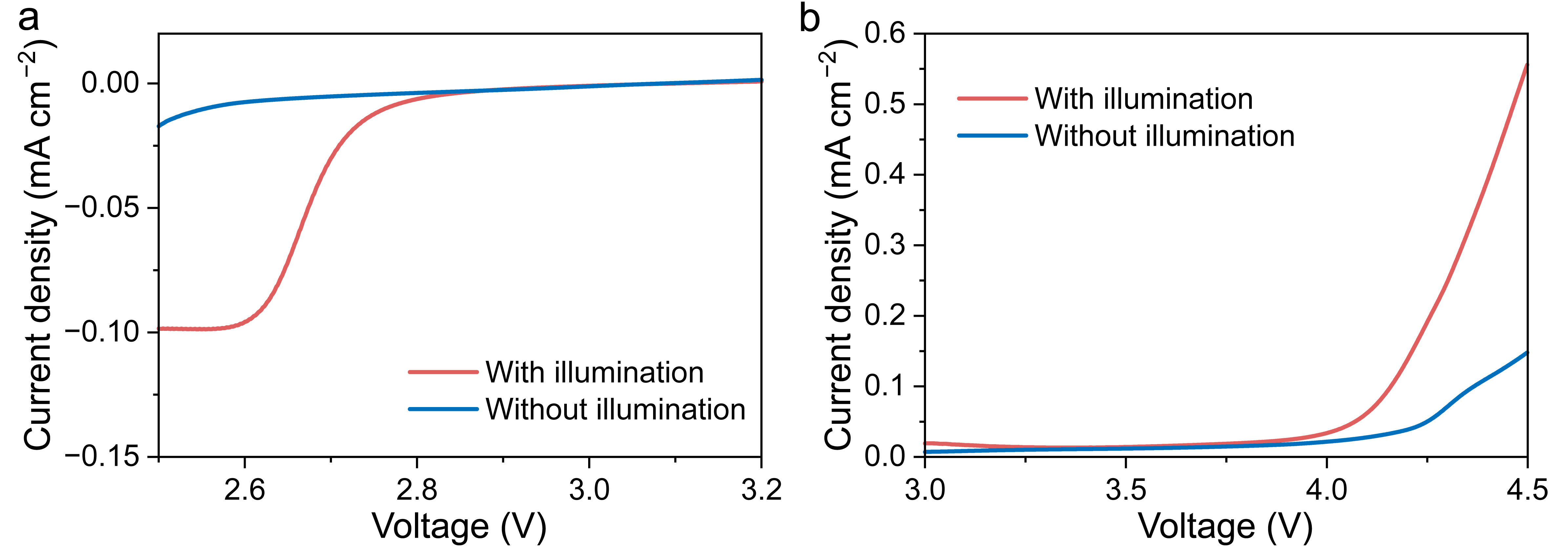
**Figure S10.** (a) ORR and (b) OER polarization curves of Bi_3_TiNbO_9_ at 1600 rpm rotating speeds with and without illumination using RRDE.

**
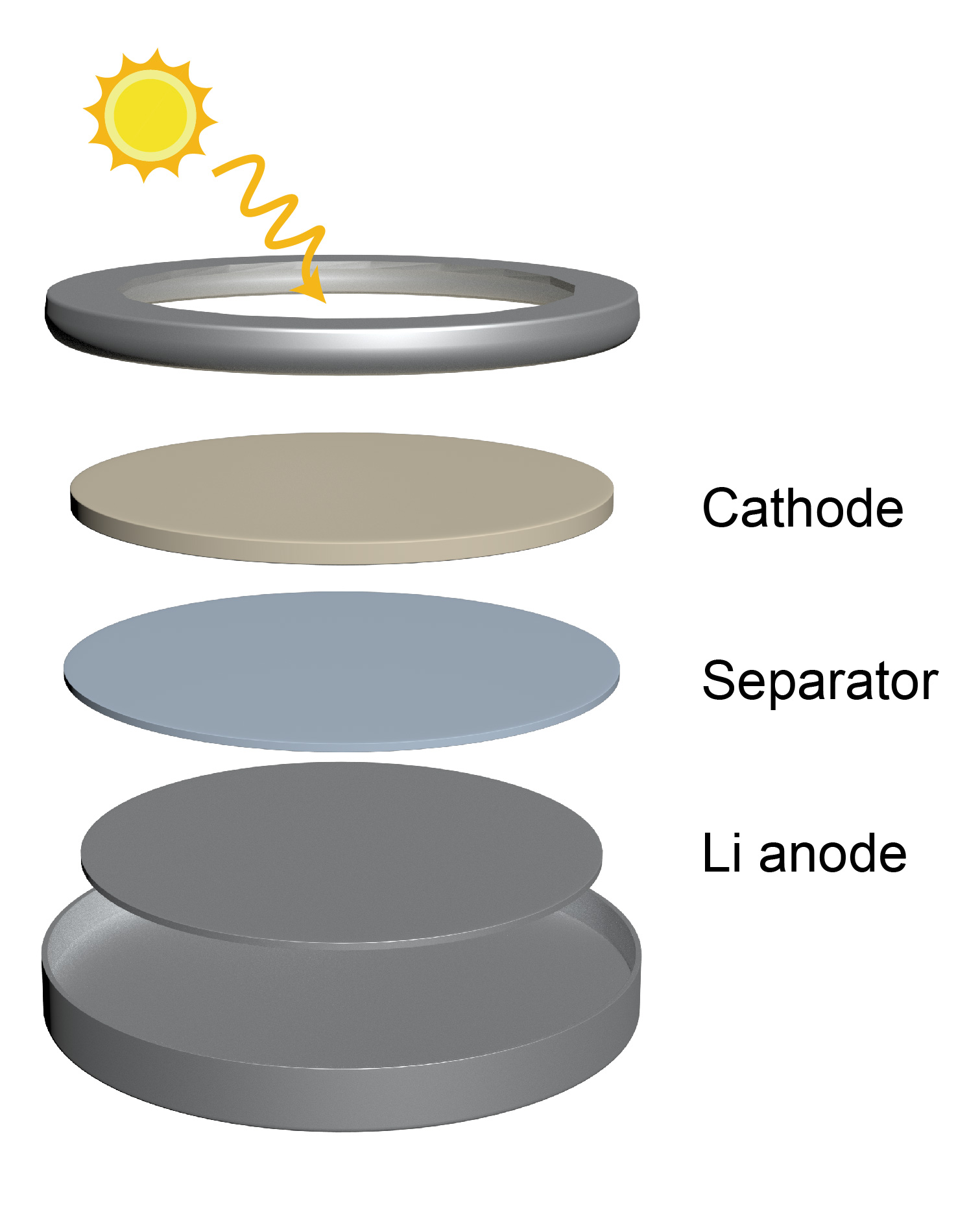
**

**Figure S11.** Schematic illustration of the structure of the photo-assisted Li–O_2_ battery.

**
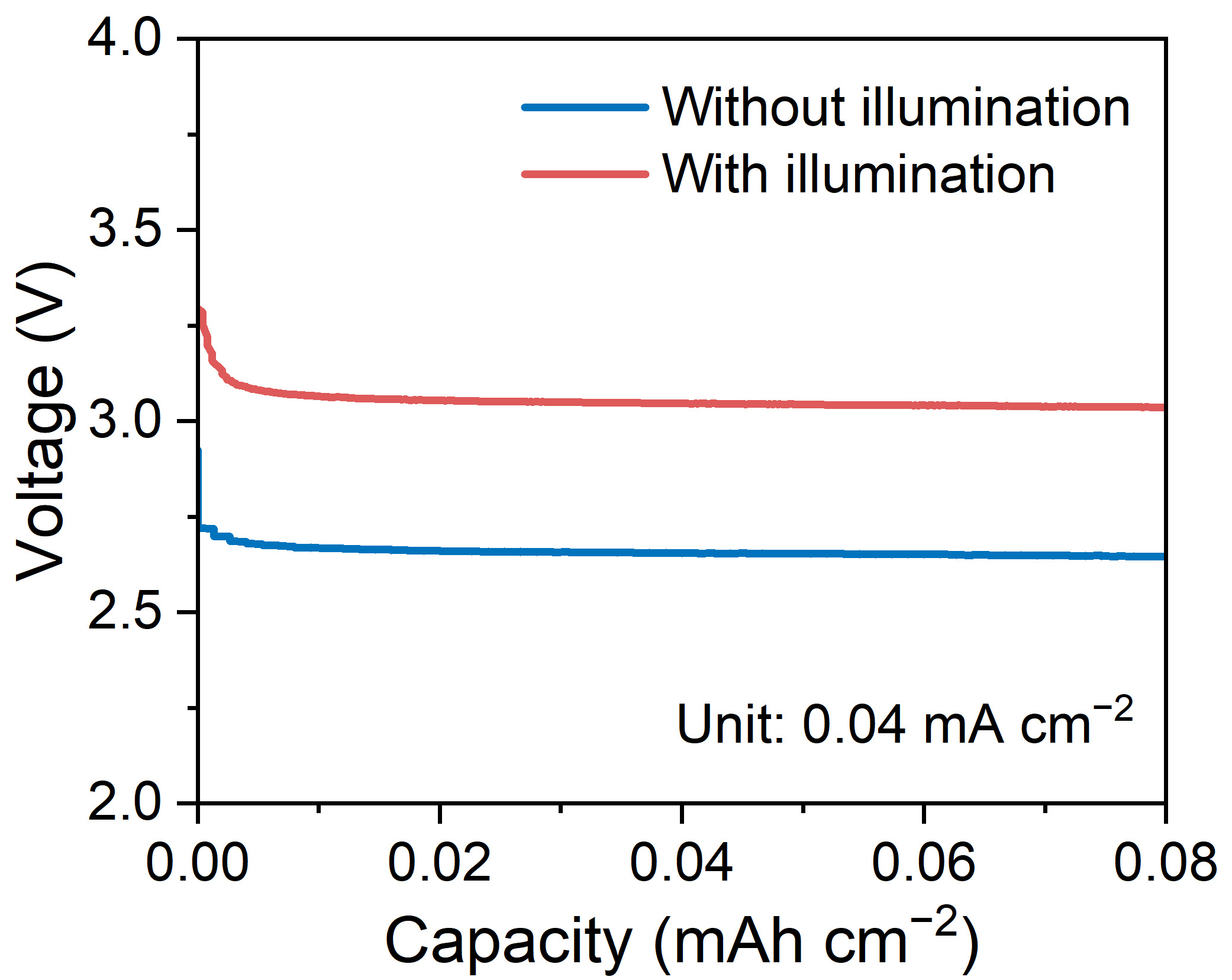
**

**Figure S12.** Discharge profiles of the Bi_3_TiNbO_9_ cathode at a current density of 0.04 mA cm^−2^ with and without illumination.

**
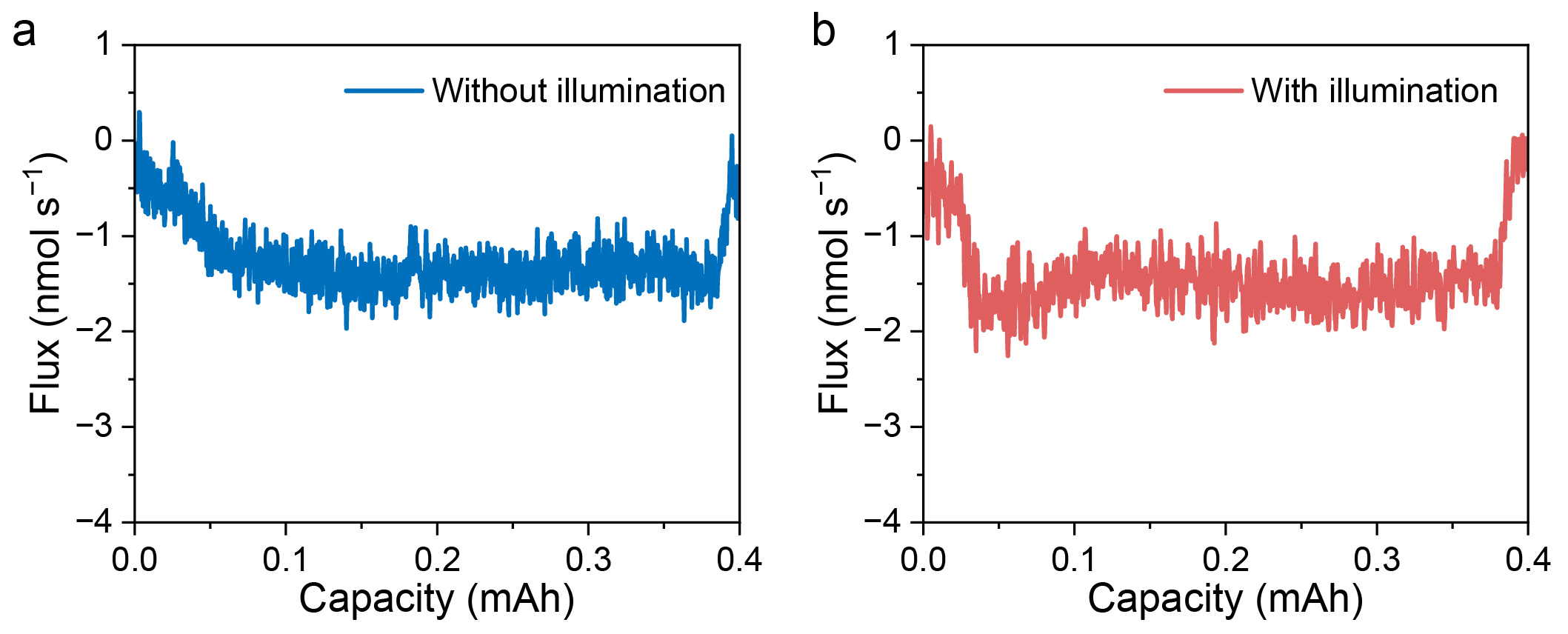
**

**Figure S13.** (a-b) The O_2_ consumption profiles of Li-O_2_ batteries with and without illumination.

**
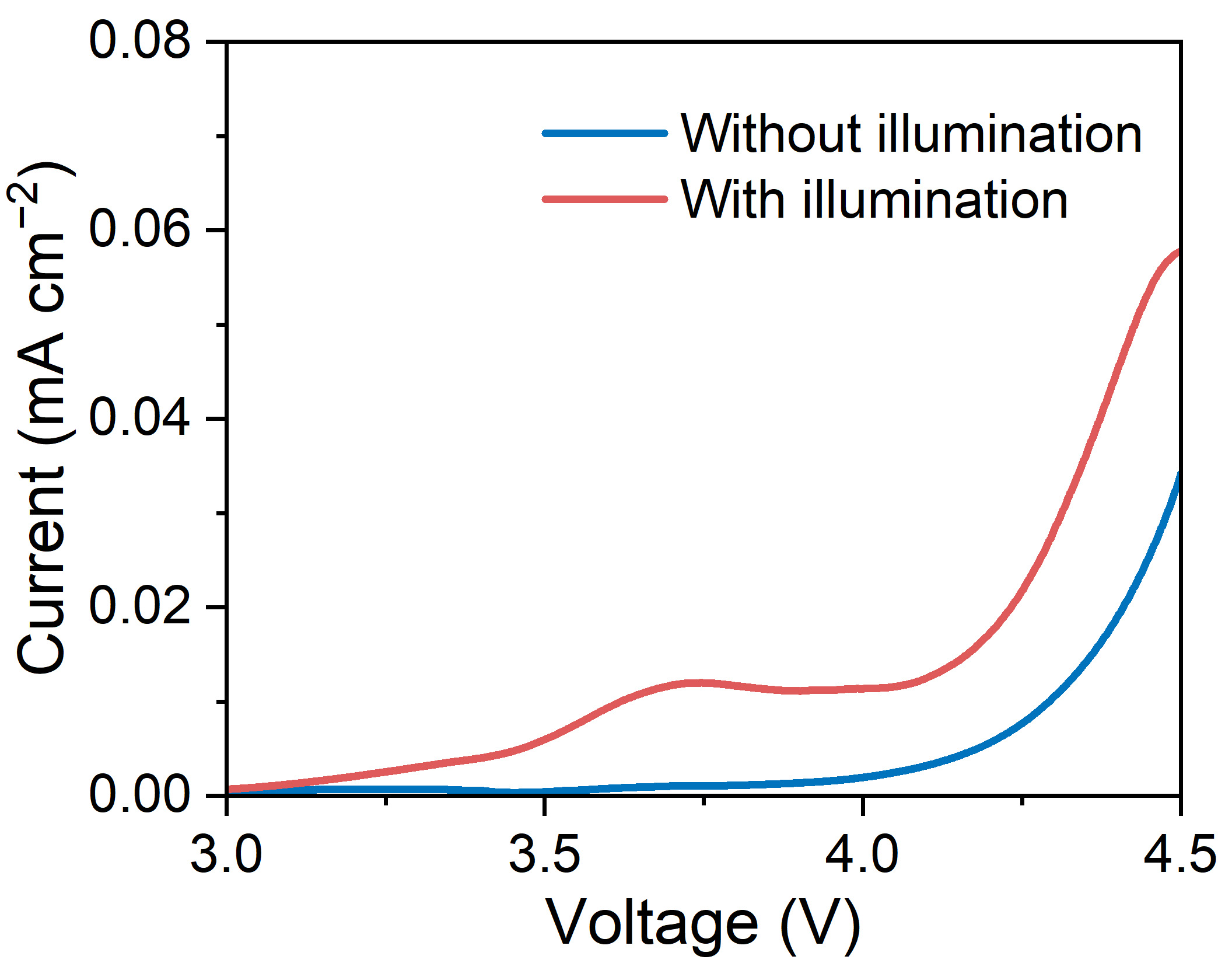
**

**Figure S14.** LSV plots of the Li–O_2_ batteries conducted with and without illumination at a scan rate of 0.1 mV s^−1^. The Li–O_2_ batteries were galvanostatically discharged for 2 h at a current density of 0.04 mA cm^−2^.


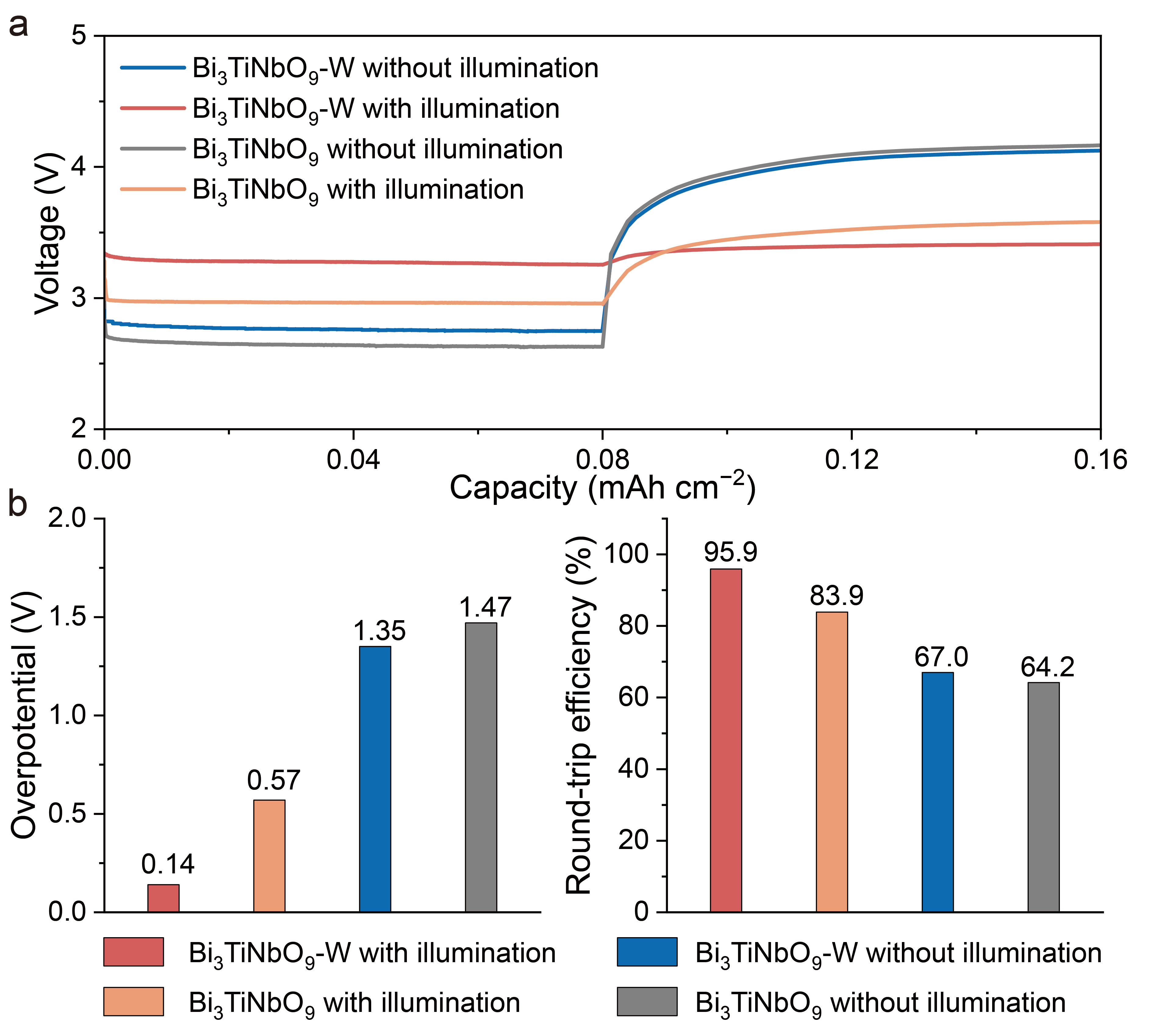


**Figure S15.** (a) Discharge/charge profiles of the Bi_3_TiNbO_9_ and Bi_3_TiNbO_9_-W cathode at a current density of 0.04 mA cm^−2^ with and without illumination. (b) Comparison of overpotential and round-trip efficiency.

**
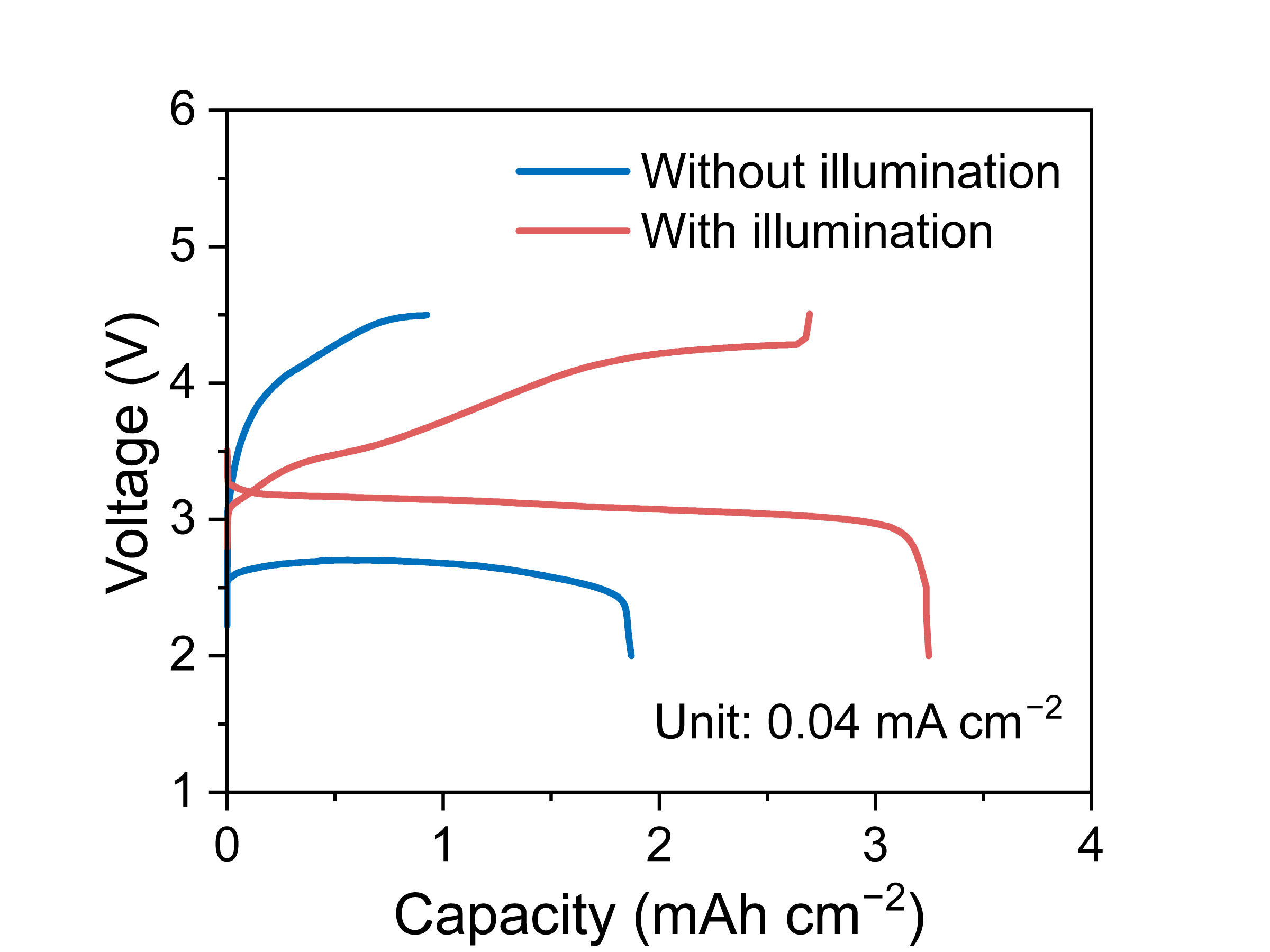
**

**Figure S16.** Discharge/charge curves of Li–O_2_ batteries based on Bi_3_TiNbO_9_-W cathode at a current density of 0.04 mA cm^−2^ in the voltage limitation range of 2.0~4.5 V with and without illumination.


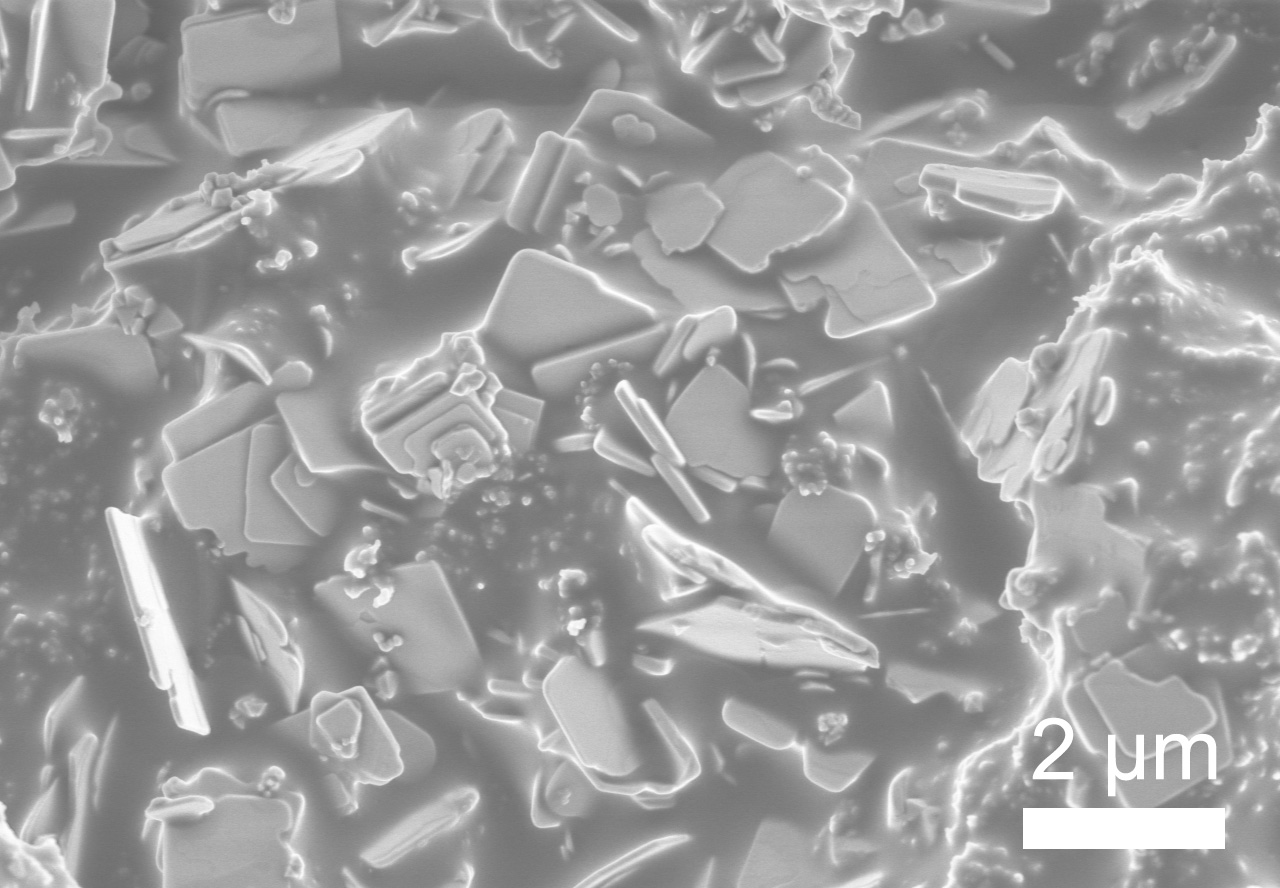


**Figure S17.** SEM images of the Bi_3_TiNbO_9_-W cathodes after the 10th charging process without illumination.

**
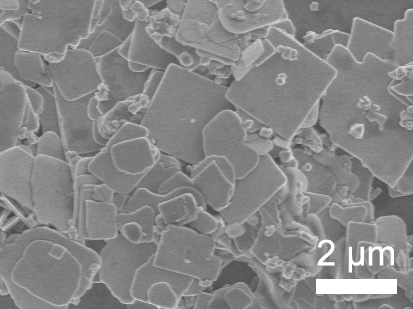
**

**Figure S18.** SEM images of the Bi_3_TiNbO_9_-W cathodes after the 10th charging process with illumination.


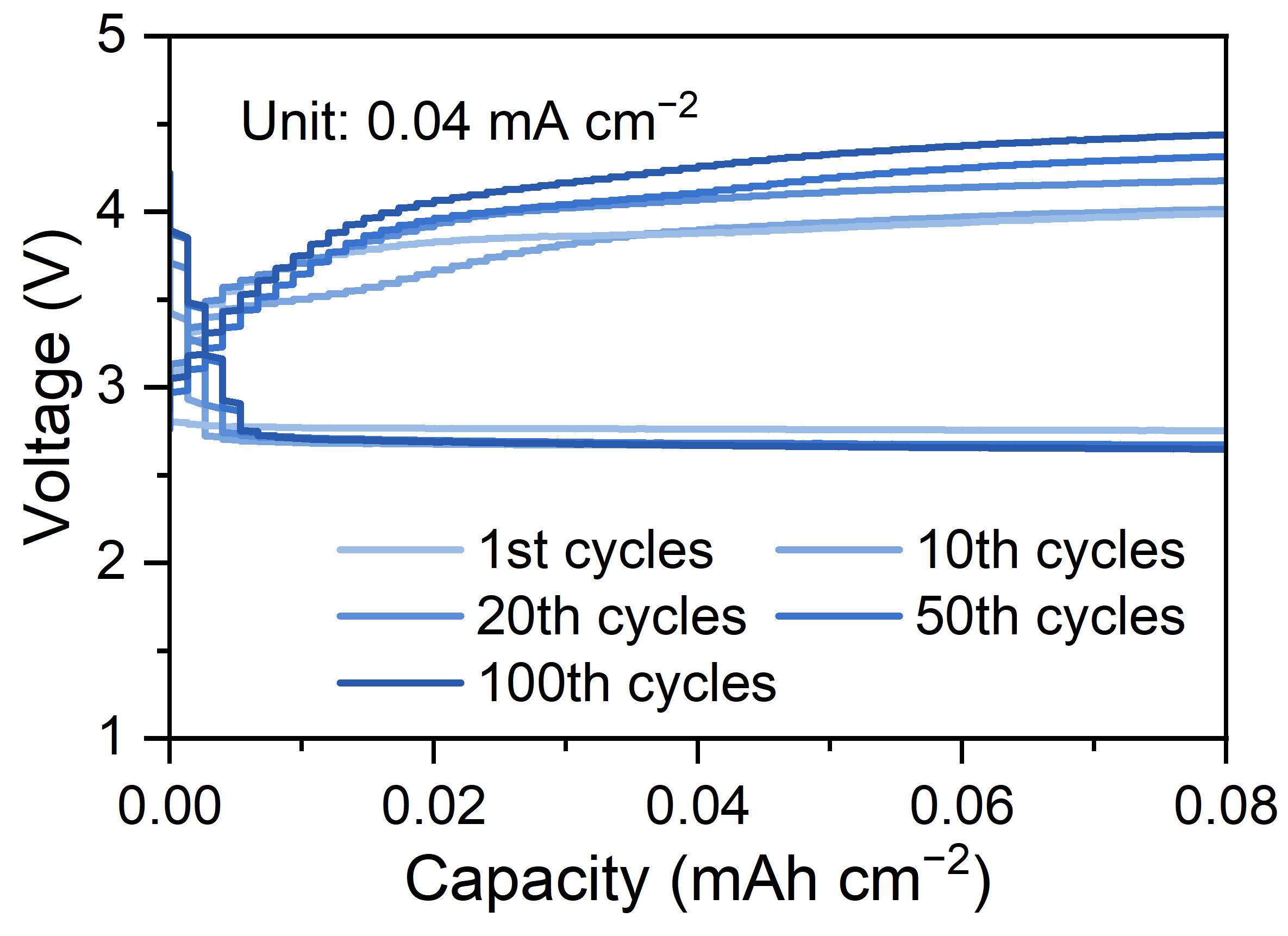


**Figure S19.** Galvanostatic discharge and charge curves of the Li–O_2_ batteries at 0.04 mA cm^−2^ without illumination for 100 cycles.

**
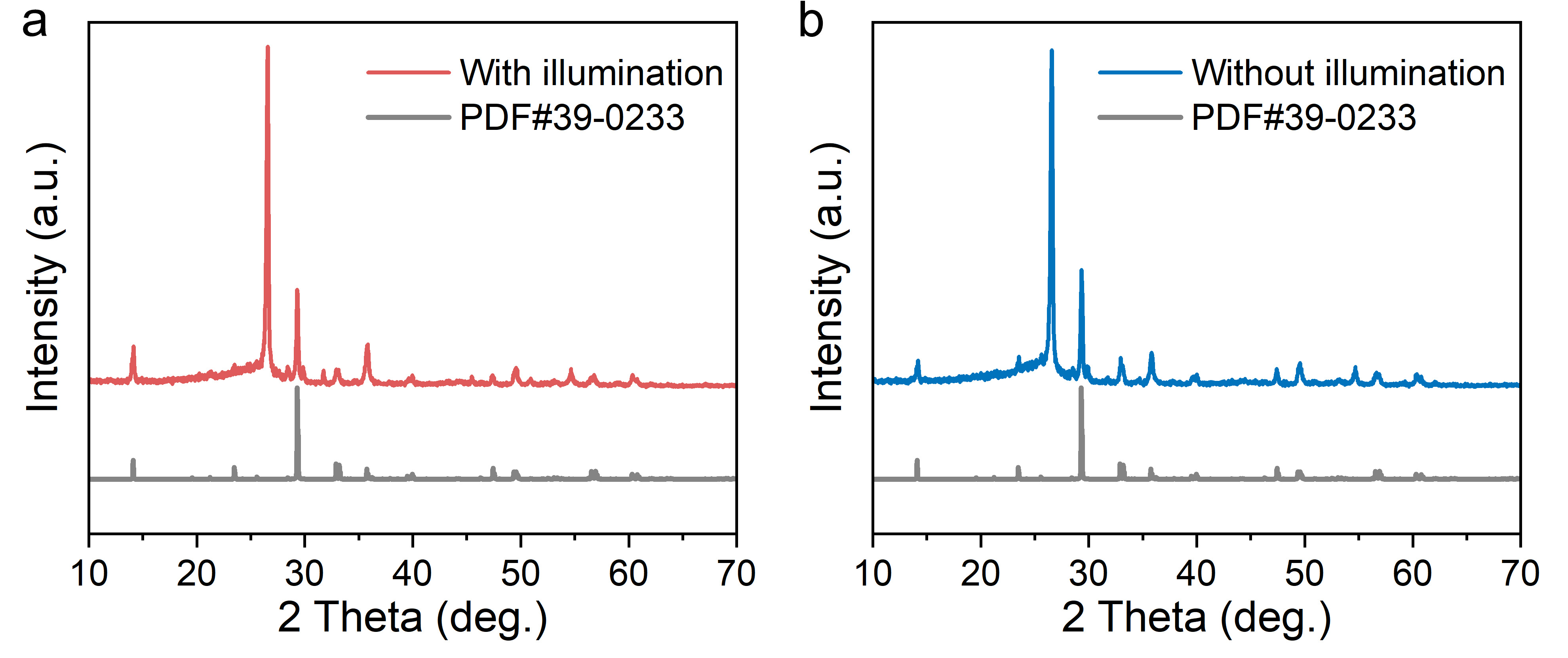
**

**Figure S20.** XRD patterns of Bi_3_TiNbO_9_-W cathodes after 10 cycles with and without illumination.

**
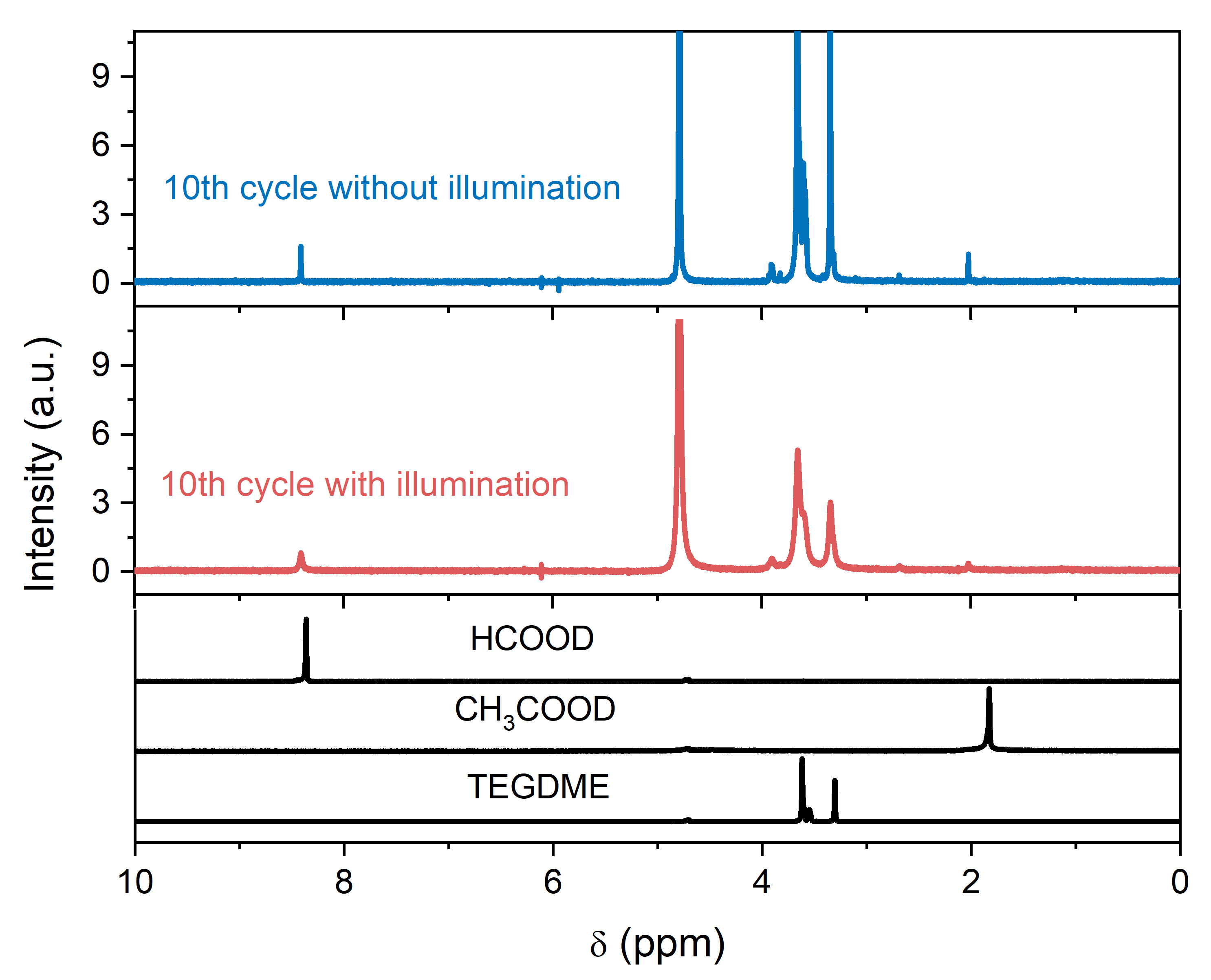
**

**Figure S21.** ^1^H NMR spectra of Bi_3_TiNbO_9_-W cathodes after the 10th cycle with and without illumination.

The battery cycled without illumination exhibited obvious lithium acetate (CH_3_COOLi) and lithium formate (HCOOLi) peaks after 10 cycles. Accordingly, although the electrolyte seems to be decomposed more easily due to the high catalytic activity under illumination, the photo-assisted charging process could effectively suppress the generation of by-products at high voltages.

**
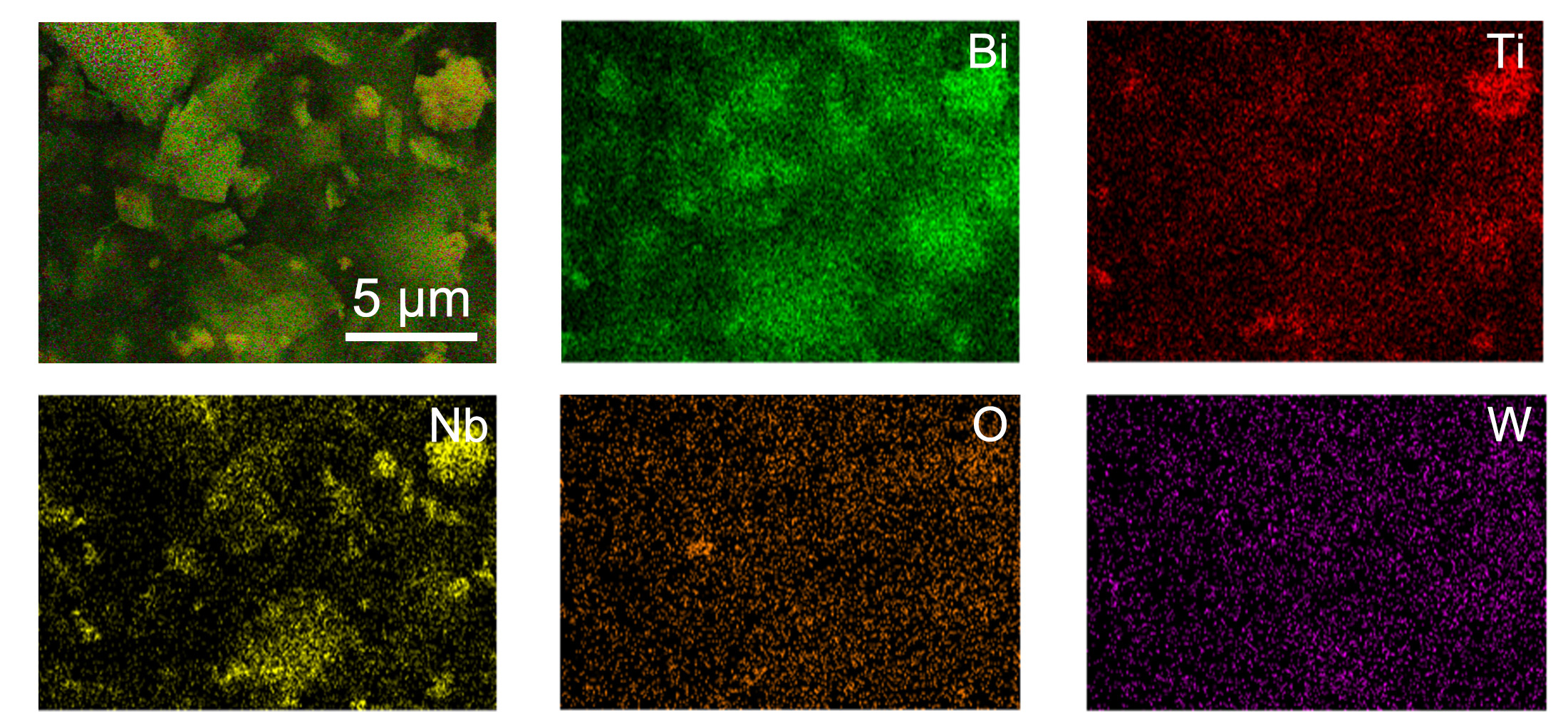
**

**Figure S22.** Element mappings of Bi_3_TiNbO_9_-W cathode after 10 cycles with illumination.


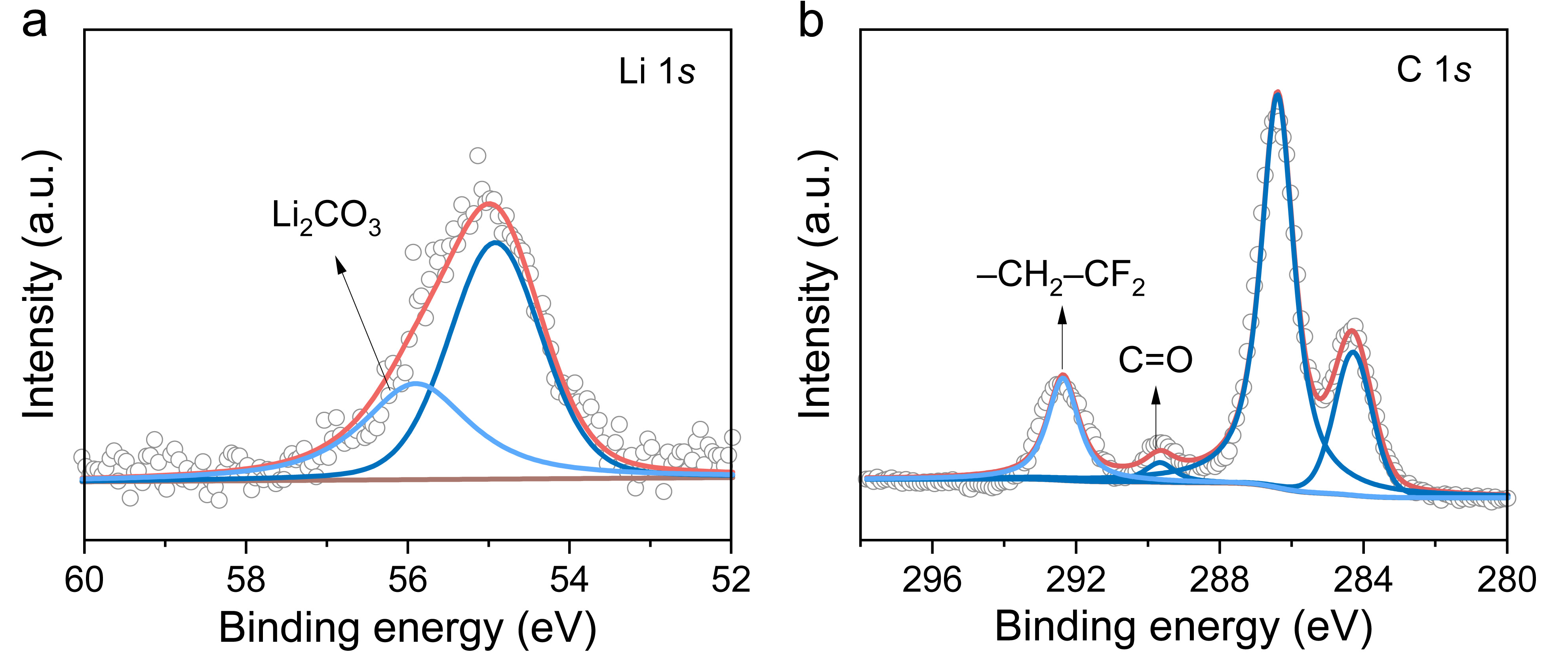


**Figure S23.** (a) Li 1*s* and (b) C 1*s* XPS spectra of the Bi_3_TiNbO_9_-W cathode after being discharged without illumination.

**Table S1**. Comparison of cycle number and initial round-trip efficiency of Bi_3_TiNbO_9_-W cathode with literature-reported Li–O_2_ batteries.

| Cathode | Cycle number | Initial round-trip efficiency (%) | Ref. |
| --- | --- | --- | --- |
| TiO_2_–Fe_2_O_3_ | 100 | 94.4 | [2] |
| C_3_N_4_ | 10 | 95.3 | [3] |
| Co-TABQ | 50 | 94.0 | [4] |
| Fe_2_O_3_/C_3_N_4_ | 50 | 98.1 | [5] |
| Fe-MOF | 195 | 93.0 | [6] |
| Ag/Bi_2_MoO_6_ | 500 h | 93.8 | [7] |
| Defective TiO_2_ | 30 | 92.6 | [8] |
| TiO_2_−Au | 200 | 86.0 | [9] |
| CsPbBr_3_@PCN-333(Fe) | 100 | 92.7 | [10] |
| (4,4′-EDP) Pb_2_Br_6_ | 170 h | 85.1 | [11] |
| pTTh | 150 | 97.0 | [12] |
| Ru nanostructures | 50 | 83.0 | [13] |
| Ag nanowires | 40 h | 65.7 | [14] |
| Ag nanocubes | 146 h | 93.9 | [14] |
| NiO nanosheets | 60 | 96.7 | [15] |
| Bi_3_TiNbO_9_-W | 240 (960 h) | 95.9 | This work |

**References**

[1] J. Huang, Y. Kang, J. Liu, T. Yao, J. Qiu, P. Du, B. Huang, W. Hu, Y. Liang, T. Xie, C. Chen, L. C. Yin, L. Wang, H. M. Cheng, G. Liu, *Nat. Commun.* 2023, **14**, 7948.

[2] M. Li, X. Wang, F. Li, L. Zheng, J. Xu, J. Yu, *Adv. Mater.* 2020, **32**, 1907098.

[3] Z. Zhu, X. Shi, G. Fan, F. Li, J. Chen, *Angew. Chem. Int. Ed.* 2019, **58**, 19021–19026.

[4] Q. Lv, Z. Zhu, S. Zhao, L. Wang, Q. Zhao, F. Li, L. A. Archer, J. Chen, *J. Am. Chem. Soc.* 2021, **143**, 1941.

[5] Z. Zhu, Q. Lv, Y. Ni, S. Gao, J. Geng, J. Liang, F. Li, *Angew. Chem., Int. Ed.* 2022, **61**, e202116699.

[6] Y. Tao, X. Fan, X. Yu, K. Gong, Y. Xia, H. Gong, H. Chen, X. Huang, A. Zhang, T. Wang, J. He, *Small* 2024, **20**, 2403683.

[7] F. Li, M. Li, H. Wang, X. Wang, L. Zheng, D. Guan, L. Chang, J. Xu, Y. Wang, *Adv. Mater.* 2022, **34**, 2107826.

[8] H. Gong, T. Wang, H. Xue, X. Fan, B. Gao, H. Zhang, L. Shi, J. He, J. Ye, *Energy Storage Mater.* 2018, **13**, 49.

[9] S. Tong, C. Luo, J. Li, Z. Mei, M. Wu, A. P. O'Mullane, H. Y. Zhu, *Angew. Chem. Int. Ed.* 2020, **59**, 20909.

[10] G. Y. Qiao, D. Guan, S. Yuan, H. Rao, X. Chen, J. A. Wang, J. S. Qin, J. J. Xu, J. Yu, *J. Am. Chem. Soc.* 2021, **143**, 14253.

[11] R. Fan, Y. Wu, H. Xie, Y. Gao, L. Wang, B. Zhao, D. Li, S. Liu, Y. Zhang, H. Kong, Y. Li, Q. Chen, A. Cao, H. Zhou, *ChemSusChem* 2022, **15**, e202201473.

[12] W. Liu, Y. Yang, X. Hu, Q. Zhang, C. Wang, J. Wei, Z. Xie, Z. Zhou, *Small* 2022, **18**, 2200334.

[13] H. Song, S. Wang, X. Song, J. Wang, K. Jiang, S. Huang, M. Han, J. Xu, P. He, K. Chen, H. Zhou, *Energy Environ. Sci.* 2020, **13**, 1205.

[14] F. Li, L. Zheng, X. Wang, M. Li, J. Xu, Y. Wang, *ACS Appl. Mater. Interfaces* 2021, **13**, 26123.

[15] X. Wang, D. Guan, F. Li, M. Li, L. Zheng, J. Xu, *Adv. Mater.* 2022, **34**, 2104792.
